# Metagenomic analysis of soybean endosphere microbiome to reveal signatures of microbes for health and disease

**DOI:** 10.1186/s43141-023-00535-4

**Published:** 2023-08-16

**Authors:** Usha Chouhan, Umesh Gamad, Jyoti Kant Choudhari

**Affiliations:** 1 Department of Mathematics, Bioinformatics & Computer Applications, Maulana Azad National Institute of Technology, Bhopal, 462051 MP India; 2 School of Biotechnology, Devi Ahilya Vishwavidyalaya, Indore, MP 452001 India

**Keywords:** Metagenomics, Endospheric mg-rast, Omicbox, Taxonomy, Function

## Abstract

**Background:**

Soil metagenomics is a cultivation-independent molecular strategy for investigating and exploiting the diversity of soil microbial communities. Soil microbial diversity is essential because it is critical to sustaining soil health for agricultural productivity and protection against harmful organisms. This study aimed to perform a metagenomic analysis of the soybean endosphere (all microbial communities found in plant leaves) to reveal signatures of microbes for health and disease.

**Results:**

The dataset is based on the National Center for Biotechnology Information (NCBI) Sequence Read Archive (SRA) release “microbial diversity in soybean”. The quality control process rejected 21 of the evaluated sequences (0.03% of the total sequences). Dereplication determined that 68,994 sequences were artificial duplicate readings, and removed them from consideration. Ribosomal Ribonucleic acid (RNA) genes were present in 72,747 sequences that successfully passed quality control (QC). Finally, we found that hierarchical classification for taxonomic assignment was conducted using MG-RAST, and the considered dataset of the metagenome domain of bacteria (99.68%) dominated the other groups. In Eukaryotes (0.31%) and unclassified sequence 2 (0.00%) in the taxonomic classification of bacteria in the genus group, *Streptomyces*, *Chryseobacterium*, *Ppaenibacillus*, *Bacillus*, *and Mitsuaria* were found. We also found some biological pathways, such as CMP-KDO biosynthesis II (from D-arabinose 5-phosphate), tricarboxylic acid cycle (TCA) cycle (plant), citrate cycle (TCA cycle), fatty acid biosynthesis, and glyoxylate and dicarboxylate metabolism. Gene prediction uncovered 1,180 sequences, 15,172 of which included gene products, with the shortest sequence being 131 bases and maximum length 3829 base pairs. The gene list was additionally annotated using Integrated Microbial Genomes and Microbiomes. The annotation process yielded a total of 240 genes found in 177 bacterial strains. These gene products were found in the genome of strain 7598. Large volumes of data are generated using modern sequencing technology to sample all genes in all species present in a given complex sample.

**Conclusions:**

These data revealed that it is a rich source of potential biomarkers for soybean plants. The results of this study will help us to understand the role of the endosphere microbiome in plant health and identify the microbial signatures of health and disease. The MG-RAST is a public resource for the automated phylogenetic and functional study of metagenomes. This is a powerful tool for investigating the diversity and function of microbial communities.

**Supplementary Information:**

The online version contains supplementary material available at 10.1186/s43141-023-00535-4.

## Background

Soybean, Glycine max (L.) Merrill is an annual, self-pollinated diploid legume (subfamily Fabaceae). Soybeans have been grown as a commercial crop, mainly in temperate ecologies, for thousands of years. One of the most widely cultivated legumes, soybeans, came initially from East Asia, but can now be found everywhere [1]. The United States has grown soybeans over the most significant areas. It accounts for approximately 32% of the world's soybean production, followed by Brazil (31%), Argentina (19%), China (6%), and India (4%) [2]. Madhya Pradesh has the highest soybean production in any other state. Soybeans have been cultivated in the Indian state of Madhya Pradesh during the last two years over an area of around 4.4 million hectares (ha), with a total yield of approximately 3.9 million tonnes and average productivity of 796–885 kg/ha [3]. The conditions under which soybeans were grown were similar to those of maize. In addition to being used to make oil, crayons, and other products, soybeans are also used for a variety of other uses. Its output is almost identical to that of maize [4]. The microbial population living in the roots of soybean plants is diverse and mostly composed of bacteria and fungi [5]. Interactions between the plant host and its microbial communities determine microbiome diversity and taxonomy, and assist critical plant activities, such as nutrient absorption and tolerance to biotic and abiotic changes [6]. Plant microbiomes may include both beneficial and harmful bacteria. Microbes inhabit the root rhizosphere and endosphere, which are composed of the outermost tissue layers of the root identified via research on experimental model plants and crops [7]. The microbial communities that are isolated from the various root compartments each have unique taxonomic structures and functional compositions [8, 9], highlighting the significance of the intricate connections between various bacterial and fungal communities and the role that these communities play in the formation of the microbiome [10]. The Microbiomes of many plant species support plant defense against pathogens and environmental stress through mechanisms such as hormone induction, nutrient absorption, and transport [11]. There has been a meteoric rise in the number of studies conducted in recent years with the objective of describing the human microbiome (the environment, including the microbiota, any proteins or metabolites they make, their metagenome, and host proteins and metabolites in this environment) in both healthy and diseased conditions. The field of microbiology has undergone a paradigm shift in the last 30 years, which has caused a change not only in our perspective on microorganisms, but also in the techniques that are used to investigate them. This has led to significant development [12]. In the early part of the twentieth century, there was a widespread belief that microbes would not exist if they could not be cultivated in a laboratory [13]. Metagenomics was first noted by [14], which encompassed information on the entire microbial community composition and function, widening the area of genomics where only genetic material is studied. A few prior studies, such as [15], on phylogenetic analyses of environmental microbial communities have also been reported [16]. In the process of metagenomics study, genomic DNA from all organisms in a community (metagenome) was extracted for fragmentation, cloning, transformation, and subsequent screening of the constructed metagenomic library. Initially, the primary target of metagenomics was limited to screening environmental communities for a specific biological activity and to identifying the related genomics [17, 18]. Although it is considered to have a significant impact in determining the outcome of metagenome analysis, it circumvents the uncolorability and genomic diversity of most microbes, the biggest roadblocks to advances in microbiology that are not properly cultured in the laboratory and identification. Knowledge gaps in understanding unculturable microorganisms and functional and taxonomic analyses are fundamental limitations [19]. Metagenomics studies can be tackled using the targeted metagenomics approach and shotgun metagenomics approach with fundamental differences based on methodology and objectives. In targeted metagenomics, a gene or a few genes are sequenced and used primarily to carry out phylogenetic studies, whereas in shotgun metagenomics, all the present DNA is sequenced and used in functional gene analysis assays (Morgan et al. 2013). This process usually involves next-generation sequencing (NGS) after DNA is extracted from the samples. This resulted in a large amount of data in the form of short reads.

In this study, we investigated the microbes present in the soybean endosphere and identified their taxonomy, function, and genes. The endosphere microbiome of soybean plants is composed of a wide variety of bacteria and fungi, which play an important role in plant health. Beneficial microbes can improve plant nutrition by increasing the availability of nutrients to plants. They can also protect plants from disease by competing with pathogens for space and nutrients, and by producing antibiotics. In addition, beneficial microbes can help plants tolerate stress by producing enzymes that detoxify the stress hormones. In this process, each piece of DNA is assigned to a particular taxonomic group, such as species, genus, or family. There are many different methods that can be used for taxonomic classification, but one of the most common is called “taxonomic hits distribution”. In the taxonomic hit distribution, the sequenced DNA was compared with a reference database of known DNA sequences. This reference database can either be a collection of known genomes or a collection of known genes. The reference database was searched for the best match to each piece of DNA in the sample, and then the taxonomic group of the reference sequence was assigned to the piece of DNA in the sample.

## Method

### Dataset acquired and processing

The SRR10740534 dataset has been retrived from the NCBI SRA and is based on the paper “microbial diversity in soybean”. Fastq-dump SRA toolkit software was used to convert the data file from the Sequence Alignment Map (SAM) to FASTQ format. Most sequencers produce sequence files in FASTQ format, which is a standard. This is similar to the FASTA format, where Q represents the quality [17]. Along with the sequence, it is recommended that the FASTQ file contain the sequence and quality of the sequence bases. The primary detail of the dataset is given in Table 1, and the basic statistics of the considered dataset is given in Table 2 below.

**Table 1:** Primary detail of the dataset

**Item**	Platform	Read Count	Base Count	Library Layout	Library Strategy	Library Source	Library Selection
SRR10740534	Illumina	80,889	44,414,941	Paired	AMPLICON	metagenomic	PCR

**Table 2:** Analysis Statistics detail of the dataset

**Upload: bp Count**	33,439,021 bp
**Upload: Sequences Count**	72,868
**Upload: Mean Sequence Length**	459 ± 18 bp
**Upload: Mean guanine-cytosine (GC) percent**	55 ± 3%
**Artificial Duplicate Reads: Sequence Count**	68,994
**Post QC: bp Count**	1,739,799 bp
**Post QC: Sequences Count**	3,853
**Post QC: Mean Sequence Length**	452 ± 39 bp
**Post QC: Mean GC percent**	55 ± 4%
**Processed: Predicted Protein Features**	5
**Processed: Predicted rRNA Features**	3,507
**Alignment: Identified Protein Features**	0
**Alignment: Identified rRNA Features**	3,507
**Annotation: Identified functional Categories**	undefined

### MG-Rast-server

We utilized MG-RAST (version 4.0.3) to control quality, predict proteins, and organize and annotate nucleic acid sequence databases. MG-RAST compares the predicted proteins to database proteins (for shotgun) and compares the 16S and 18S sequences to reads. MG-RAST allows access to phylogenetic and metabolic reconstructions [20, 21].

### Data processing

We selected the following pipeline for data processing.

1. *Assembled*: If the file contains assembly data, we choose the assembled input sequence option and include coverage information within each sequence header.
2. *Dereplication*: This process includes removing artificially replicated sequences that are artificially processed.
3. *Screening*- There is a filter for hot species in screening, and then we select the specific species. It removes any host species sequence, for example, plant, human, mouse, and others, with the help of DNA-level matching with a bowtie [22].
4. *Dynamic trimming*: This method removes low-quality sequences using dynamic cutting.

### Omics Box tools

Omics Box is bioinformatics software that converts readings into insights. For each collection of sequences, these tools enable the identification of pathways, function analysis, gene prediction, and other functions from multiple databases [23]. The OmicsBox tools were used to predict gene function, pathway, and gene modules.

### IMG/M

IMG/M is an integrated genome and metagenome comparative data analysis system that allows open access interactive analysis of publicly available datasets, whereas manual curation, submission, and access to private datasets and computationally intensive workspace-based analysis require login/password access to its expert review (ER) companion system (IMG/MER)  [24]. The core data model underlying IMG allows recording the primary sequence information and its organization in scaffolds and/or contigs [25]. Metagenome bins can be stored in IMG as individual workspace scaffold datasets, and analyzed using many tools, such as function profiles [24]. The new Scaffold Search under the Find Genomes menu provides two search modes: Quick Search allows querying of scaffolds in IMG using scaffold IDs, while Advanced Search allows querying of scaffolds using various metadata attributes [26].

## Result and discussion

### Sequencing quality analysis

**Fig. 1:**
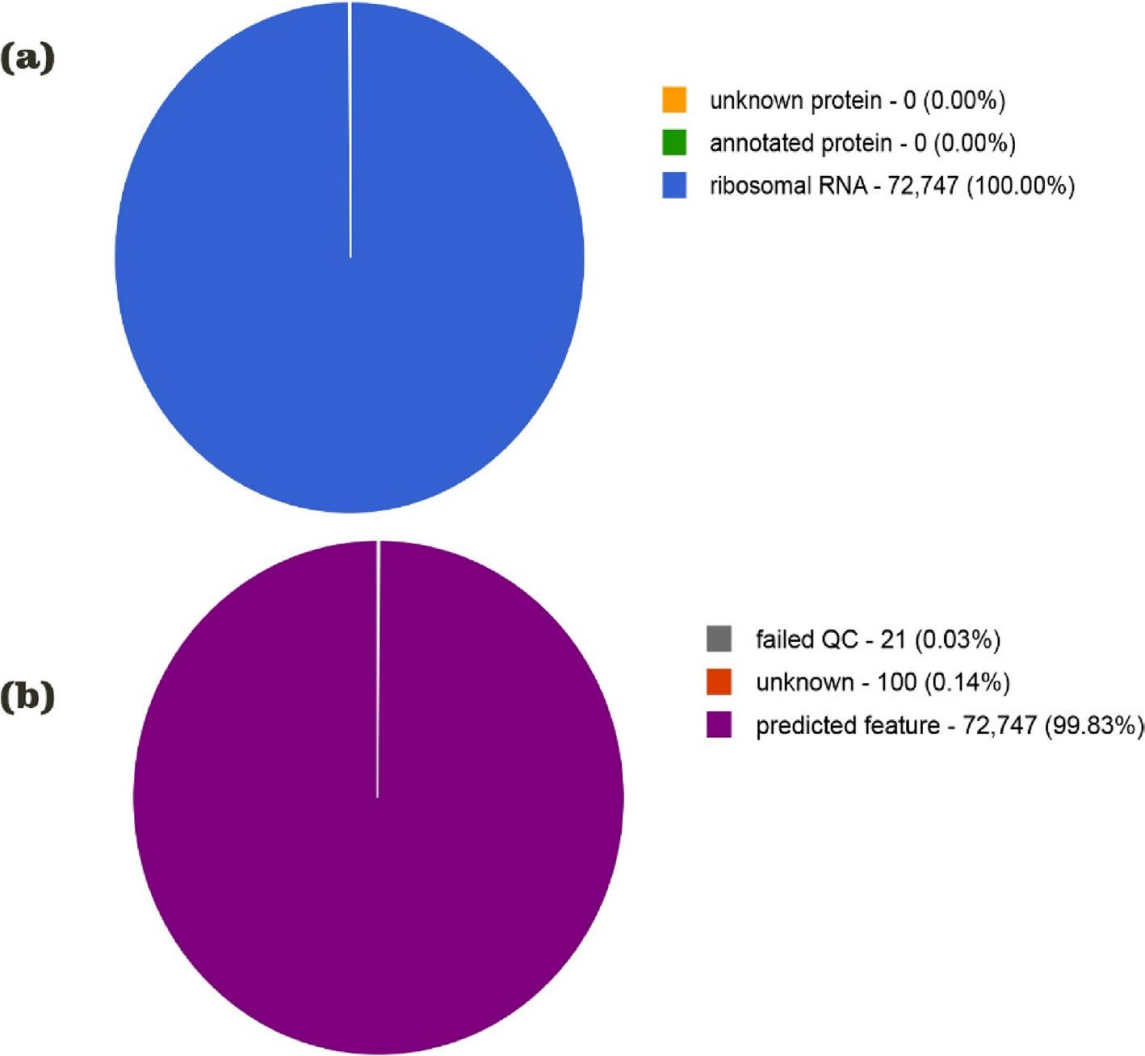
QC result of Sequences **a** Sequence Breakdown **b** Predicted Features

To process the metagenome data analysis, dataset quality analysis was performed using the FastQC program. The FastQC program provides a QC report on spot problems that originate either in the sequencer or in the starting library material. Many modules were used to evaluate the raw data, and an HTML report with a module summary was created. The pre-alignment steps are specified in the quality control report. Run the FastQC summary report and compare the read format information with the overall poor quality to filter out and cut low-quality sequence parts while maintaining high-quality sequences. The dataset contained 72,868 sequences, totalling 203,552,249 base pairs (bp) with an average length of 378 ± 77 bp. The quality control process rejected 21 of the evaluated sequences (0.03% of the total), as shown in Fig. 1. Dereplication determined that 68,994 sequences were artificial duplicate readings, and removed them from consideration. Ribosomal RNA genes were present in each of the 72,747 sequences that successfully passed the QC. In Fig. 1(a, b), the feature breakdown and function of the QC are shown.

### Source hits distribution

**Fig. 2:**
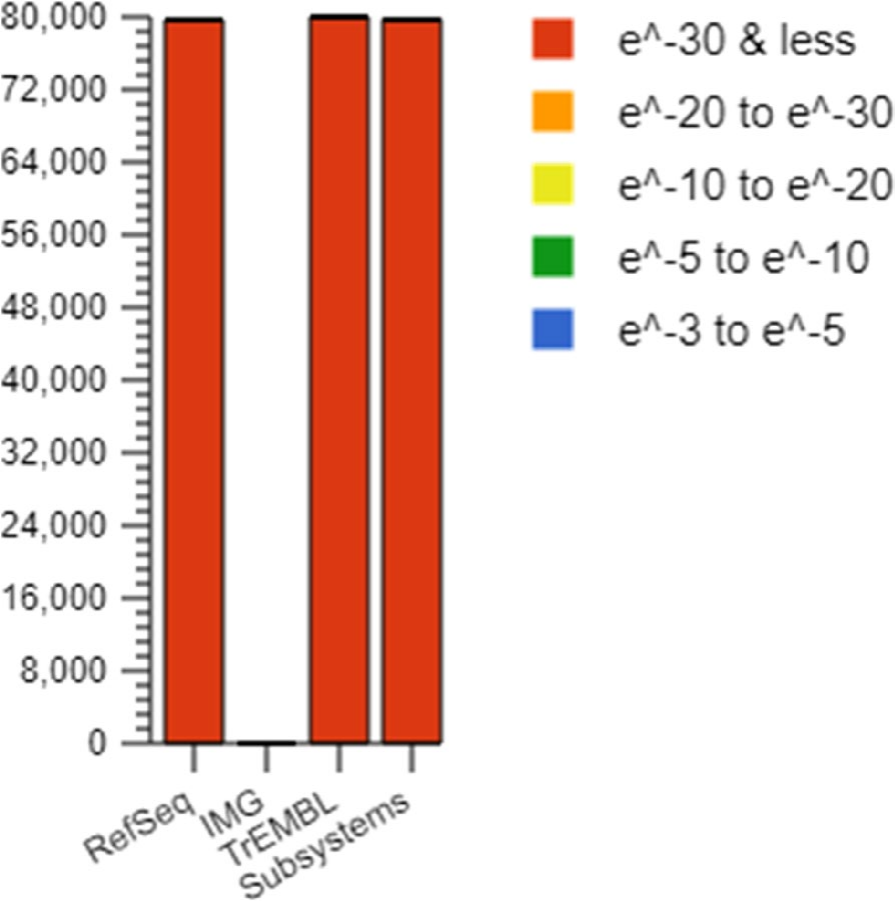
Source hit distribution of studied data set

The biological interpretation of the source hit distribution is essential for providing information on how many sequences per dataset were found for each database. The source hists distribution has been investigated, we have found 16 hit databases including protein databases, protein databases with functional hierarchy information, and ribosomal RNA databases with maximum in RefSeq [27], TrEMBL [28], and Subsystems shown in Fig. 2. In the figure, the bars representing annotated reads are colored based on the e-value range. It is important to note that different databases may have varying numbers of hits and can also provide different types of annotation data.

### Sequence GC distribution

**Fig. 3:**
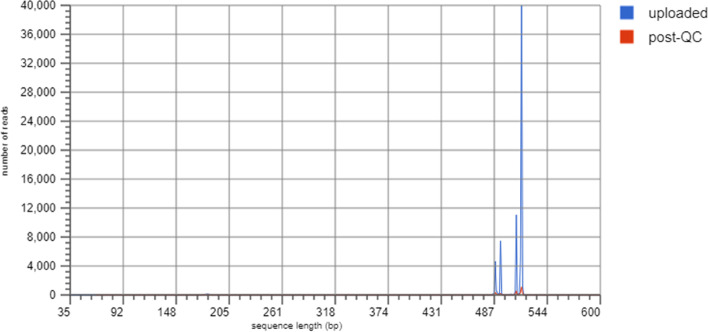
Sequence Length Histogram

**Fig. 4:**
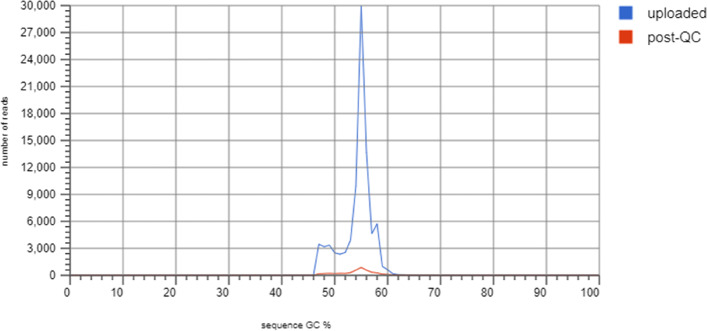
Sequence GC Distribution

The Sequence GC Distribution was evaluated as illustrated in Figs. 3 and 4. Histograms depict the sequence lengths in bp for this metagenome. Each position represents the number of base pairs (bp). The charts used raw upload and post-QC data.

In Sequence GC Distribution analysis, Guanine and Cytosine-rich areas were identified to predict the annealing temperature. Figure 4 shows the GC % distribution in the metagenome. Each location indicates the range of the GC %. The plots used raw uploaded and post-QC data.

### Taxonomic analysis

#### Taxonomic hits distribution

**Fig. 5:**
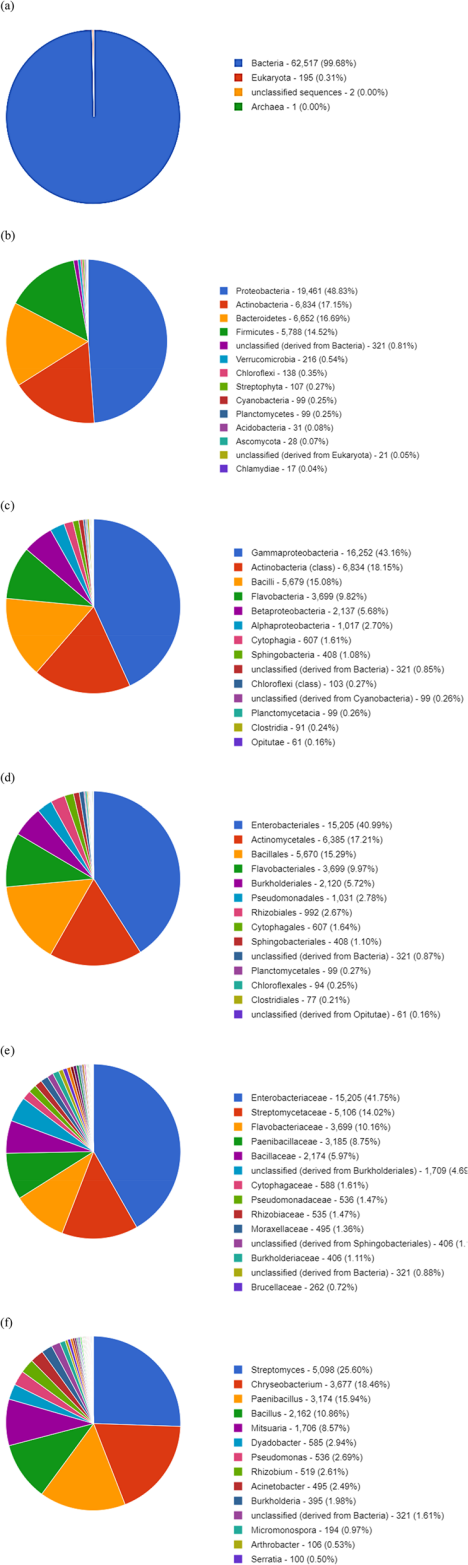
We present taxa using contig LCA to determine a single consensus taxonomic item for each sequence feature. **A** domain **B** Phylum **C** Class **D** Order **E** Family **F** Genus

When conducting a metagenomic study, one of the key parameters that is often considered is taxonomic hit distribution. This provides insights into the species present in a given sample and their relative abundance. Taxonomic hit distribution can provide insights into the species present in a given sample and their relative abundance. This information can be used to help understand the ecology of a sample and can be used to help guide future studies. Hierarchical classification for taxonomic assignment was conducted using MG-RAST, and the considered dataset of the metagenome domain of Bacteria (99.68%) dominated other groups *of eukaryotes* (0.31%) and unclassified sequence 2(0.00%). The charts below represent the distribution of taxa using a contig LCA algorithm, finding a single consensus taxonomic entity for all features on each individual sequence. Similarly, *Proteobacteria* dominated over *Actinobacteria*, B*acteroidetes*, and *Firmicutes*. In terms of bacterial taxonomy, the richest classes, orders, families, and genus are as follows *Streptomyces* (25.60%), *Chryseobacterium* (18.46%), *Paenibacillus* (15.94%), *Bacillus* (10.86%), *Mitsuaria* (8.57%), *Dyadobacter* (2.94%), *Pseudomonas* (2.69%), *Rhizobium*, (2.61%) *Acinetobacter* (2.49%), *Burkholderia*, (1.98%) unclassified (derived from Bacteria)- (1.6), *Micromonospora* (0.97%), *Arthrobacter* (0.53%), *Serratia*—(0.50%) These percentages indicate the prevalence of each taxonomic entity, as depicted in Fig. 5(a-f). The distribution of taxa was determined using a contigLCA algorithm, which assigned a single consensus taxonomic classification to all features found in each individual sequence. Within the realm of bacterial taxonomy, the genus Streptomyces stands out by claiming a substantial portion, approximately 25.60%, of its corresponding taxonomic category. Notably, this genus exhibits remarkable capabilities as it produces antibiotics with efficacy against a wide range of biological adversaries, including fungi, bacteria, and parasites [29]. Streptomyces has harnessed its capabilities to develop immunosuppressants and biocontrol agents specifically designed for agricultural purposes. These antibiotics exhibit the power to regulate and combat fungi and parasites, effectively safeguarding crops like soybeans from potential damage caused by these microorganisms. Moreover, Streptomyces demonstrates its prowess by suppressing or eradicating microbial adversaries, while simultaneously stimulating plant growth in various agricultural settings. This remarkable phenomenon has been observed across multiple crop types, leading to significant improvements in soybean crop production. Furthermore, the presence of Streptomyces contributes to the overall promotion and enhancement of soybean crop growth [30]. Among the recorded sequences, it was determined that the genus *Corynebacterium* held the second-highest percentage, amounting to approximately 18.46%. The majority of Gram-positive bacteria falling under this classification exhibit the ability to thrive and persist in oxygen-rich environments. *Corynebacterium* species are ubiquitous, inhabiting the soil layers on the skin of mammals. According to the genetic characteristics, examination of the Corynebacterium genome revealed both harmful and non-pathogenic species [31]. Within the genus, Paenibacillus emerges as the third prominent species, accounting for a substantial percentage of approximately 15.94%. This particular species comprises a multitude of sequences that play a pivotal role in facilitating the growth and development of soybean crops [32]. Paenibacillus species that establish a symbiotic relationship with plants possess the remarkable ability to produce auxin phytohormones, which exert a profound influence on plant development. In addition to this, these species facilitate the uptake of phosphorus by plant roots and some of them even engage in atmospheric nitrogen fixation, thereby providing substantial benefits to soybean plants. Furthermore, Paenibacillus plays a crucial role in suppressing phytopathogens through the production of biocides that contribute to systemic resistance [33]. *Bacillus* emerges as the third prominent species, accounting for a substantial percentage of approximately 10.86%, *Bacillus* species are predominately found in food and have both beneficial and harmful effects on human health. This is because these microbes produce bioactive substances during fermentation. Consequently, eating food made from soybeans fermented by *Bacillus* ensures food safety [34]. Additionally, *Bacillus* helps the seedlings of soybean plants to become more resistant to infection. Bacillus species possess a wide variety of advantageous tracts. Plants benefit from this process because they can obtain nutrients. Increased synthesis of phytohormones leads to better overall growth and increased resistance to both biotic and abiotic stressors [34]. There are numerous varieties of bacteria found in this genus, which may be advantageous and helpful for soybean plant development and metabolic activity, and some aid as a biofertilizer and biotic and abiotic stress, and have a major function in soybean crops. Within the context of soybean plants, the genus *Mitsuaria* assumes a noteworthy position. It is worth mentioning that the sequences affiliated with *Mitsuaria* constitute the fourth largest segment, amounting to approximately 8.57% sequence. Mitsuaria isolates have been observed to inhibit fungal and oomycete plant pathogens in laboratory and in vivo experiments on soybean seedlings, leading to a reduction in disease severity. This study indicates the effectiveness of T-RFLP-derived markers for identifying microorganisms with pathogen-inhibiting properties [35]. The metagenomic sequences reveal that several genera, including Pseudomonas, Rhizobium, Burkholderia, and Mesorhizobium, co-exist in the rhizosphere and nodules of soybean plants [36]. Additionally, endophytic bacteria, including *Burkholderia*, *Rhizobium*, *Bradyrhizobium*, *Mesorhizobium*, and *Dyadobacter*, have been identified as beneficial for plant growth and development. Some studies have also investigated the effects of plant growth promoting rhizobacteria (PGPR) on soybean growth and soil bacterial community composition. For example, Paenibacillus *mucilaginosus*, a PGPR strain, improved symbiotic nodulation, soybean growth parameters, nutrient contents, and yields in a field experiment [37]. The certain genus *Acinetobacter*, *Micromonospora*, and *Serratia* species in the soybean metagenome promote plant growth through nitrogen fixation, phosphate solubilization, siderophore production, phytohormone synthesis, and enhanced tolerance to salinity. Their presence and activities contribute to the overall growth and development of soybean plants.

#### Rank abundance plot

**Fig. 6:**
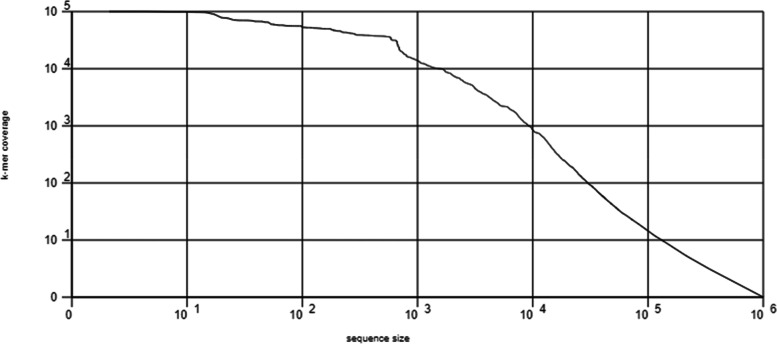
kmer rank abundance graph plots the kmer coverage as a function of abundance rank

To graphically depict taxonomic richness and evenness, Rank Abundance plots were arranged the taxonomic abundances in descending order from their most abundant to their least abundant values. In most cases, only the top 50 most prevalent cases are presented. On a logarithmic scale, the abundance of annotations is shown along the y axis. The most abundant sequences on the left are shown in Fig. 6.

#### Rarefaction curve

**Fig. 7:**
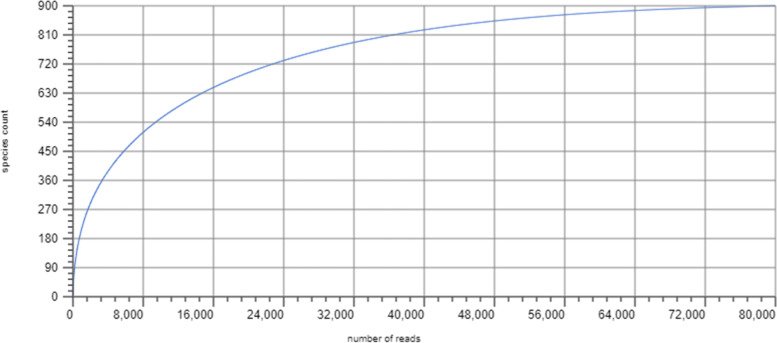
Rarefaction Curve showing the richness of the annotated species

The rarefaction curve shows the total number of different species annotations as a function of the number of sampled sequences. This curve indicates the richness of the annotated species (Fig. 7).

### Scaffold analysis from the genome sequence

**Fig. 8:**
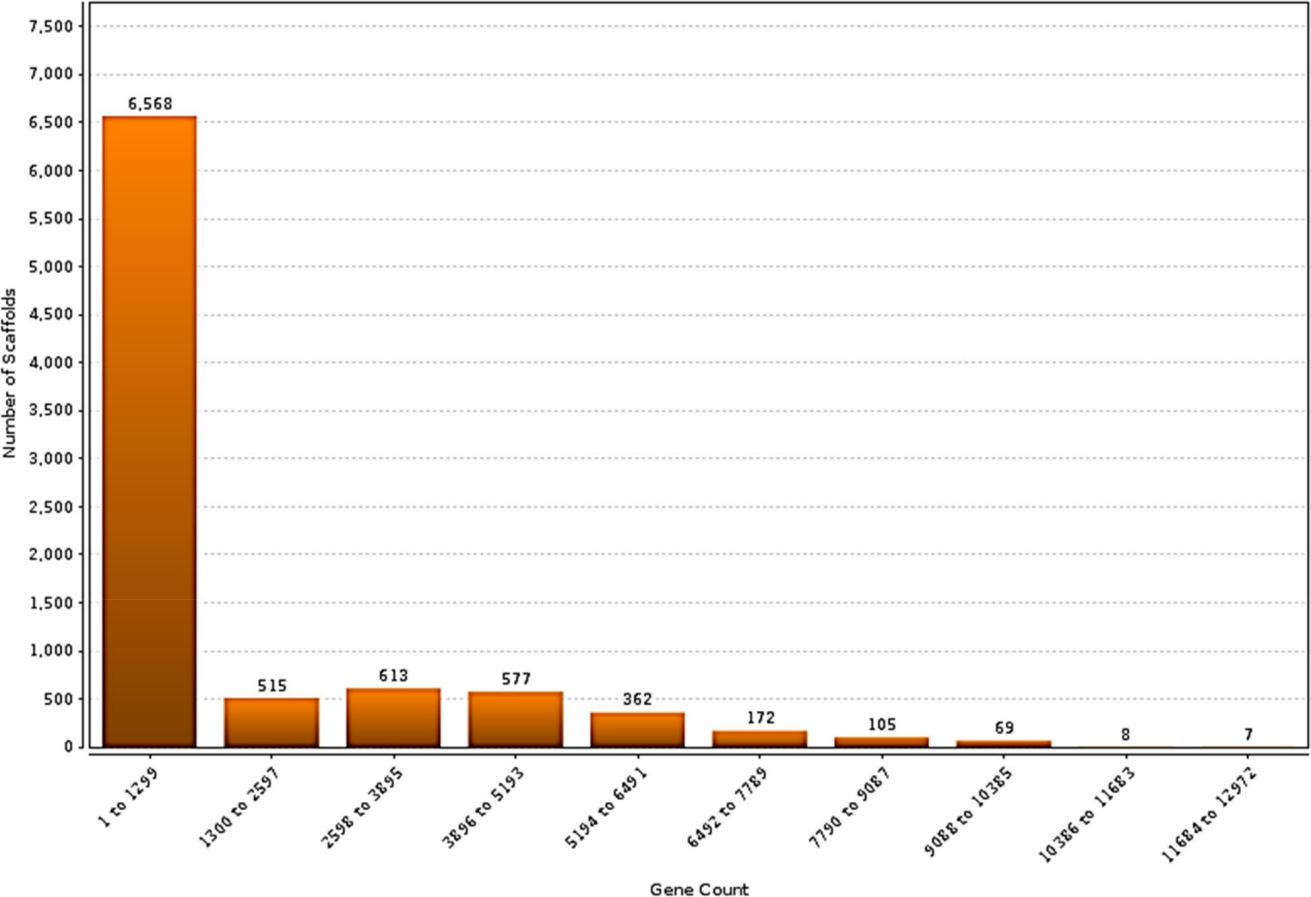
Scaffold analysis. Scaffolds by Gene Count Histogram

**Fig. 9:**
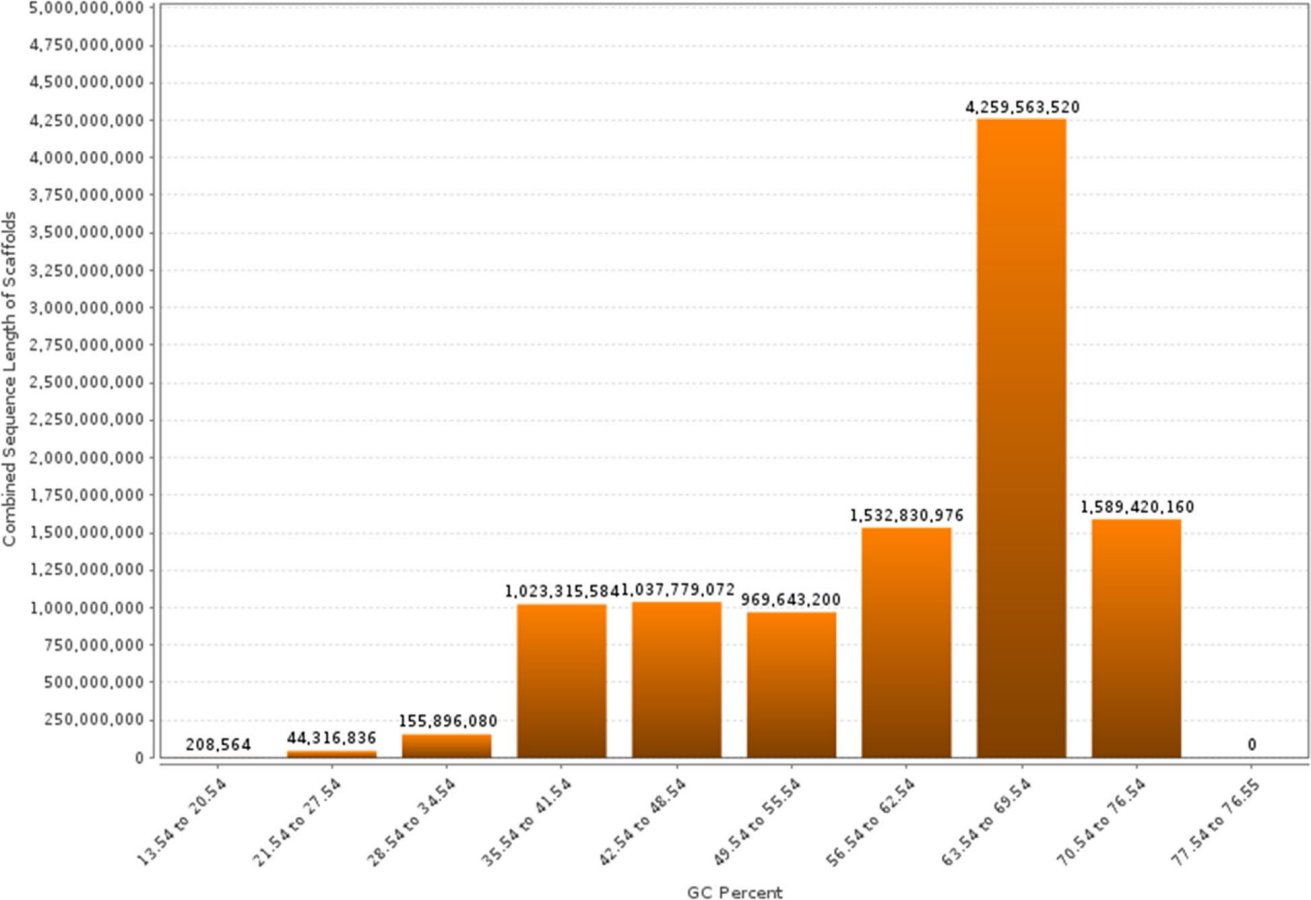
Scaffold analysis. Scaffolds by GC Percent Histogram

A scaffold is a reconstructed genomic sequence from whole-genome shotgun clones, consisting of contigs and gaps. It is created by chaining contigs together and separating them by gaps. Whole-genome shotgun assembly aims to represent each genomic sequence in one scaffold, but it is not possible. Scaffolding improves the contiguity and quality of metagenomic bins by assembling short metagenomic reads into longer contiguous sequences based on sequence overlap. The distribution of scaffolds by gene count provided valuable insights into the prevalence and distribution patterns of genes within the metagenomic dataset. The analysis revealed varying numbers of scaffolds within specific gene count ranges, indicating varying levels of gene abundance and representation. The histogram tab in Scaffold Cart displays a histogram with the counts of protein-coding genes in the sample. Analysis of gene count distribution within metagenomic datasets plays a fundamental role in unraveling the complexity and functional diversity of microbial communities. In this study, the distribution of scaffolds by gene count was thoroughly examined, providing valuable insights into the prevalence and distribution of genes across a metagenomic dataset. The results demonstrated a comprehensive breakdown of scaffolds falling within specific gene count ranges, ranging from 1 to 12,972, as shown in Fig. 8. This detailed breakdown enabled a deeper understanding of the distribution patterns and relative abundance of genes within the metagenomic dataset. By elucidating the number of scaffolds within each gene count range, this analysis sheds light on the genetic composition and functional potential of microbial communities, thereby contributing to our knowledge of the intricate dynamics of these complex ecosystems. In this study, we identified 6,568 scaffolds, with gene counts ranging from 1 to 1,299. Of these, 515 scaffolds had gene counts ranging from 1,300 to 2,597; 613 scaffolds had gene counts ranging from 2,598 to 3,895; 577 scaffolds had gene counts ranging from 3,896 to 5,193; 362 scaffolds had gene counts ranging from 5,194 to 6,491; 172 scaffolds had gene counts ranging from 6,492 to 7,789; 105 scaffolds had gene counts ranging from 7,790 to 9,087; 69 scaffolds had gene counts ranging from 9,088 to 10,385; eight scaffolds had gene counts ranging from 10,386 to 11,683; and seven scaffolds had gene counts ranging from 11,684 to 12,972. The distribution of gene counts across scaffolds was nonuniform, with a higher proportion of scaffolds having fewer genes. This suggests that the genome is composed of a large number of small genes and a smaller number of larger genes. The distribution of gene counts may also be influenced by the assembly method used because different methods may have different biases in the number of genes that can be detected. The identification of scaffolds with different numbers of genes is important for understanding genome organization. Genes are often clustered together on scaffolds, and the number of genes on a scaffold can be used to infer their functions. For example, scaffolds with a large number of genes are often associated with metabolic pathways, whereas scaffolds with a small number of genes are often associated with regulatory functions. In addition to analyzing the gene count distribution, it is essential to examine other relevant parameters that provide further insights into the metagenomic dataset. This section focuses on the GC percentage, scaffold count, and combined sequence length. These parameters contribute to our understanding of the composition and structural characteristics of the datasets. Figure 9 presents the distribution of scaffolds based on their GC percentage range along with the corresponding scaffold count and combined sequence length. This analysis provided an overview of the dataset and presented the values for each GC percentage range. In the range of 13.54 to 20.54, there were 208,564 scaffolds with a combined sequence length of 208,564 base pairs. In the range of 21.54 to 27.54, we observed a significant increase in scaffold count, with 44,316,835 scaffolds and an equivalent combined sequence length. Similarly, the ranges of 28.54 to 34.54, 35.54 to 41.54, 42.54 to 48.54, 49.54 to 55.54, 56.54 to 62.54, 63.54 to 69.54, and 70.54 to 76.54 show varying scaffold counts and combined sequence lengths. These values provide valuable insights into the composition and characteristics of the metagenomic dataset, offering a quantitative representation of the genomic content within each GC percent range. This helps to unravel the dataset's characteristics, genomic diversity, and structural properties of the microbial communities under study.

### Function analysis

OmicsBox is a bioinformatics software platform that enables researchers to go from raw data to meaningful insights within a couple of hours [23]. Functional studies of the Clusters of Orthologous Groups (COG), cellular activities and signalling, metabolism, and storage were carried out. The analysis was carried out on 827 sequences, each of which had an average length of 44.0 characters. Only 1.81% of the sequences had gene ontology (GO) annotations, leading to the discovery of 75 GO term annotations. Functional analysis of COG considers that, in the instance of Metabolic Processes, the predominant functions by sequence are amino acid translation, ribosomal structure and biogenesis, and replication, recombination, and repair. Metagenomic analysis of the fermented soybean product sikkam indicated that the sequence activities include translation, ribosomal structure and biogenesis, replication, recombination, and repair [38]. Mapping of metagenomic sequences against databases of orthologous gene groups revealed many enriched recombination and repair functional sequences.

### Gene prediction

Gene prediction is an important tool in metagenomics and in the study of the genetic material of an entire ecosystem. By examining the genes of organisms in a sample, scientists can learn about the functions of these genes and the organisms themselves. There are several methods of gene prediction, but they all center on one basic process: looking for regions of the genome that are likely to encode proteins. Proteins are the building blocks of all living organisms; therefore, genes encoding proteins are often the most important. There are many different types of proteins, each with a specific function. Some proteins are involved in metabolism, whereas others are involved in cell structure regulation. Regardless of the function of the protein, gene prediction can help to identify the genes that encode it. By identifying these genes, scientists can learn about the function of the proteins and the organisms that produce them. Gene prediction uncovered 1,180 sequences, 15,172 of which included gene products, with the shortest sequence being 131 bases and maximum length 3829 base pairs. These gene products were found in the genome of strain 7598. The maximum genes were discovered in *Bacillus, Pseudomonas, Paenibacillus, Klebsiella, Lactobacillus, Streptomyces, Bradyrhizobium, Bordetella, Cronobacter, Salmonella, Corynebacterium, Micromonospora, Cupriavidus, Akkermansia, Leuconostoc, Xanthomonas, Priestia, Ligilactobacillus, and Candidatus* (Table S1). The gene list was additionally annotated using Integrated Microbial Genomes and Microbiomes. The annotation process yielded a total of 240 genes found in 177 bacterial strains, as depicted in Table 3.

**Table 3:** The gene list annotation using the metagenomic database

Genome Name	Gene Symbol	Length (bp)	Genome Name	Gene Symbol	Length (bp)
Corynebacterium glutamicum ATCC 21831	zupT	792	Marivirga tractuosa H-43, DSM 4126	mutS2	2397
Bacillus licheniformis DSM 13 Goettingen	yyaS	606	Frankia inefficax EuI1c	murA	1260
Bacillus licheniformis DSM 13 Goettingen	ywrA	537	Brevibacillus laterosporus NRS 682, LMG 15441	mtrB	240
Bacillus licheniformis 9945A	ywnC	393	Desulfovibrio vulgaris vulgaris DP4	mtnA	1053
Paenibacillus polymyxa CICC 10580	yvbA	312	Acidipropionibacterium acidipropionici F3E8	msrA	630
Paenibacillus sp. lzh-N1	yutG1	498	Bacillus paralicheniformis Bac48	msrA	546
Bacillus paralicheniformis Bac48	yunB	777	Modestobacter marinus BC501	mscS	837
Bacillus paralicheniformis Bac48	yugT	1683	Bacillus paralicheniformis Bac84	mrgA	465
Bacillus licheniformis DSM 13 Novozymes	yueI	402	Enterobacter cloacae A1137	mreD	489
Paenibacillus polymyxa M1	ytvI3	1155	Thioalkalivibrio paradoxus ARh 1	mrcB	2268
Streptomyces sp. RJA2910	ytnA	1434	Bradyrhizobium sp. BM-T	moaE	468
Bacillus paralicheniformis 14DA11	yrrS	693	Bacillus paralicheniformis Bac84	moaE	495
Bacillus paralicheniformis 14DA11	yrhB	1143	Paenibacillus kribbensis AM49	moaA1	1014
Paenibacillus polymyxa J	yqfU	945	Thermoanaerobacter sp. X514	mnmE	1383
Bacillus paralicheniformis MDJK30	ypzA	267	Trichodesmium erythraeum IMS101	mnmA	1080
Bacillus paralicheniformis 14DA11	ylbO	606	Xanthomonas albilineans XaFL07-1	mltD	1212
Paenibacillus polymyxa SQR-21	ykkC3	342	Klebsiella pneumoniae pneumoniae RJF293	mioC	441
Klebsiella pneumoniae pneumoniae RJF293	yjhQ	552	Priestia megaterium SF185	minJ	1191
Paenibacillus polymyxa SC2	yjbR	348	Sphingopyxis sp. MG	mfeA	903
Bacillus licheniformis 9945A	yhzE	87	Nitrosospira multiformis Nl14	mfd	3468
Paenibacillus polymyxa E681	yfmM	1581	Halobacillus halophilus HL2HP6	metN2	1053
Paenibacillus polymyxa J	yfiF5	786	Corynebacterium terpenotabidum Y-11 Genome sequencing	metI	702
Paenibacillus polymyxa J	yetL3	510	Phaeobacter inhibens P88	metF	870
Bacillus licheniformis 5NAP23	yesE	420	Bacillus sp. FJAT-21351	metB	1146
Lelliottia nimipressuralis SGAir0187	yejK	1008	Ligilactobacillus acidipiscis ACA-DC 1533	menB	822
Paenibacillus polymyxa Sb3-1	yclD	462	Enterobacter cloacae A1137	mdoG	1602
Bacillus paralicheniformis BL-09	ycgN	1551	Amphibacillus xylanus NBRC 15112	mcsB	1068
Klebsiella pneumoniae pneumoniae RJF293	ycgB	1533	Deinococcus gobiensis I-0, DSM 21396	map	744
Moorena producens PAL-8–15-08–1	ycf27	729	Cutibacterium acnes AE1	map	855
Paenibacillus polymyxa SC2	ybbK	462	Bacillus paralicheniformis 14DA11	manP	1944
Caldanaerobacter subterraneus MB4	XylB	1077	Paenibacillus sp. lzh-N1	M1-957	789
Geodermatophilus turciae DSM 44513	xylA	1185	Corynebacterium stationis ATCC 21170	lysC	1266
Paenibacillus polymyxa Mc5Re-14	xkdM	408	Bacillus sp. IHB B 7164	lysC	1233
Xanthomonas albilineans GPE PC73	XCC3509	972	Agromyces sp. AR33	Lxx16240	321
Caldicellulosiruptor bescii RKCB130	valS	2625	Methylobacillus flagellatus KT	lpxA	783
Trichormus variabilis NIES-23	valS	3045	Xanthomonas albilineans HVO005	lptC	567
Chloracidobacterium thermophilum B	valS	2685	Vitis vinifera PN40024	LOC100257077	1054
Candidatus Mycoplasma haemolamae Purdue	valS	2508	Vitis vinifera PN40024	LOC100244859	1192
Caldicellulosiruptor bescii RKCB121	valS	2625	Rhodococcus jostii RHA1	lipA	930
Cupriavidus sp. NP124	uvrD3	2094	Anabaenopsis circularis NIES-21	leuS	2640
Rudanella lutea DSM 19387	uvrB	2022	Caldanaerobacter subterraneus MB4	LepA	1812
Corynebacterium crudilactis JZ16	ureG	618	Spiribacter vilamensis DSM 21056	lepA	1824
Actinoplanes sp. SE50/110	ureB	408	Lactobacillus delbrueckii lactis KCCM 34717	Ldb2189	843
Stigmatella aurantiaca DW4/3–1	uppP	894	Lactobacillus delbrueckii jakobsenii ZN7a-9	Ldb1360	2235
Chloracidobacterium thermophilum B	uppP	855	Lactobacillus delbrueckii lactis KCTC 3035	Ldb1010	1002
Paenibacillus kribbensis AM49	ugpB5	1305	Lactobacillus delbrueckii jakobsenii ZN7a-9	Ldb0854	1254
Ochrobactrum sp. MYb15	ubiE	792	Lactobacillus delbrueckii lactis KCTC 3035	Ldb0761	234
Rhodopseudomonas palustris TIE-1	trxC	438	Cupriavidus taiwanensis LMG 19424	kynU	1257
Rhodococcus sp. MTM3W5.2	trxB	990	Micromonospora aurantiaca ATCC 27029	kptA	543
Solidesulfovibrio magneticus RS-1	truB	969	Micromonospora aurantiaca DSM 45487	kptA	543
Collimonas arenae Ter282	trpS2	1035	Paenibacillus polymyxa CICC 10580	kinA	1740
Leisingera caerulea DSM 24564	trpE	1512	Laribacter hongkongensis HLHK9	kcy	666
Brevibacillus laterosporus B9	troA	963	Phaeobacter inhibens P72	iscA	360
Pedobacter ginsengisoli T01R-27	trmD	678	Nostoc sp. Moss6	invB	1452
Geobacillus subterraneus KCTC 3922	topA	2076	Ligilactobacillus salivarius salivarius UCC118	infC	525
Candidatus Protochlamydia amoebophila UWE25	tolA	1068	Ligilactobacillus salivarius salivarius UCC118	infA	219
Mycoplasmopsis fermentans PG18	tmk	663	Phaeobacter gallaeciensis P11	hutG	786
Virgibacillus sp. SK37	tilS	1389	Candidatus Symbiobacter mobilis CR (contamination screened)	htpG	2013
Virgibacillus halodenitrificans PDB-F2	thyA	957	Kutzneria albida DSM 43870	hrcA	1023
Janthinobacterium lividum NCTC 8661	thrS	1908	Candidatus Protochlamydia amoebophila UWE25	hisH	594
Xanthomonas albilineans GPE PC73R	thrS	1905	Sphingopyxis sp. YR583	hisH	609
Arcobacter sp. L	thrS	1809	Streptomyces sp. Wb2n-11	hisG	858
Bacillus paralicheniformis MDJK30	thiM	810	Geobacter metallireducens GS-15	hisE	330
Mycobacterium bovis BCG Tokyo 172	thiL	1002	Corynebacterium stationis ATCC 21170	hisE	264
Deferribacter desulfuricans SSM1	thiH	1119	Micromonospora viridifaciens DSM 43909	hisE	264
Phaeobacter inhibens P80	thiB	978	Moorella thermoacetica ATCC 39073	hisC	1161
Candidatus Saccharimonas aalborgensis	tgt	1248	Xanthomonas albilineans FIJ080	hflD	615
Enterobacter ludwigii P101	tesB	861	Phaeobacter gallaeciensis P128	hemN1	1356
Corynebacterium striatum NCTC 9755	tcsR4	723	Corynebacterium variabile DSM 44702	hemB	1038
Corynebacterium striatum 216	tcsR3	639	Bacillus licheniformis DSM 13 Novozymes	hemA	1362
Bacillus sp. H15-1	tasA	795	Caldicellulosiruptor changbaiensis CBS-Z	hcp	1650
Xanthomonas albilineans HVO005	suxR	1032	Paenibacillus polymyxa CICC 10580	guxA	2154
Streptomyces sp. 57	sseA	840	Cupriavidus metallidurans CH34	gtrA	447
Paenibacillus polymyxa SC2	srrA1	1275	Geobacter sulfurreducens KN400	GSU2289	1446
Enterobacter cloacae A1137	srlE	960	Geobacter sulfurreducens PCA	GSU0207	201
Priestia megaterium WSH-002	spoIIIAD	384	Geobacter sulfurreducens KN400	GSU0201	2172
Phaeobacter piscinae P71	SPO1017	756	Phaeobacter inhibens P92	grxC	258
Sphaerobacter thermophilus 4ac11, DSM 20745	smpB	483	Rubrobacter radiotolerans RSPS-4	grpE	708
Xanthomonas albilineans GPE PC17	Smlt3089	936	Novosphingobium pentaromativorans US6-1	groS	315
Ligilactobacillus salivarius GJ-24	smc	3537	Singulisphaera sp. GP187	greA	480
Paenibacillus kribbensis AM49	sigW11	549	Xanthomonas albilineans GPE PC17	gpmA	750
Priestia megaterium WSH-002	sigI	720	Thioalkalivibrio sp. ALRh	gmhA	600
Nostoc sp. Moss3	serS	1281	Priestia megaterium Q3	gltB	1482
Actinoplanes sp. N902-109	selA	1257	Xanthomonas albilineans GPE PC17	glpK	1500
Comamonas sp. 26	secF	957	Streptomyces viridosporus T7A, ATCC 39115	glpD2	1656
Sulfurospirillum deleyianum 5175, DSM 6946	secE	180	Nostoc sp. PCC 7107	gloB	774
Phaeobacter piscinae P42	secA	2700	Xanthomonas albilineans REU209	glnB2	339
Phaeobacter piscinae P18	scpA	792	Xanthomonas sacchari LMG 476	glmU	1368
Lysinibacillus sp. YS11	scpA	780	Caldicellulosiruptor bescii MACB1021	glmS	1836
Streptomyces sp. RJA2910	SCO5669	930	Paenibacillus polymyxa M1	gldF	723
Streptomyces noursei ATCC 11455 Genome sequencing	SCO5590	591	Actinoplanes sp. N902-109	glcA	1248
Streptomyces sp. 3214.6	SCO5167	729	Geobacillus sp. C56-T3	GK3260	1290
Streptomyces viridosporus T7A, ATCC 39115	SCO0254	738	Geobacillus sp. 12AMOR1	GK3216	963
Sorangium cellulosum So ce 56	sce9191	993	Geobacillus kaustophilus HTA426	GK3038	1371
Sorangium cellulosum So ce 56	sce0166	909	Geobacillus sp. 12AMOR1	GK3036	900
Geobacillus kaustophilus HTA426	SAM	109	Geobacillus vulcani PSS1	GK2801	1860
Mycobacterium bovis BCG Tokyo 172	Rv0075	1173	Geobacillus kaustophilus Et7/4	GK2160	186
Geobacter sulfurreducens AM-1	ruvA	600	Geobacillus thermoleovorans FJAT-2391	GK1813	486
Klebsiella pneumoniae FDAARGOS_127	rsxA	582	Geobacillus vulcani PSS1	GK1582	699
Nostoc sp. PCC 7524	rsgA	1062	Geobacillus sp. GHH01	GK1316	1146
Hydrogenobaculum sp. 3684	rpsZ	189	Geobacillus sp. GHH01	GK1185	885
Mageeibacillus indolicus UPII9-5	rpsU	174	Geobacillus vulcani PSS1	GK1101	789
Micromonospora citrea DSM 43903	rpsT	267	Geobacillus kaustophilus HTA426	GK0983	675
Priestia megaterium DSM 319	rpsS	279	Geobacillus thermoleovorans FJAT-2391	GK0603	657
Spiribacter curvatus UAH-SP71	rpsP	270	Geobacillus kaustophilus Et7/4	GK0572	249
Caldicellulosiruptor bescii RKCB122	rpsP	246	Geobacillus kaustophilus HTA426	GK0545	369
Methylobacillus flagellatus KT	rpsN	306	Geobacillus kaustophilus HTA426	GK0418	225
Acidobacteriaceae sp. KBS 146	rpsK	423	Geobacillus sp. LC300	GK0324	1233
Xanthomonas albilineans XaFL07-1	rpsG	468	Anaeromyxobacter sp. Fw109-5	gcvT	1083
Phaeobacter gallaeciensis P73	rpsF	354	Candidatus Endomicrobium trichonymphae Rs-D17	gcvPB	1437
Sphingopyxis terrae ummariensis UI2	rpsE	714	Geobacillus subterraneus KCTC 3922	gcvH	384
Agromyces sp. 23–23	rpsC	753	Cutibacterium acnes PA_15_1_R1	gcvH	372
Bacillus licheniformis DSM 13 Goettingen	rpoZ	201	Corynebacterium striatum 216	gatA	1485
Desulfovibrio vulgaris vulgaris DP4	rpoD	1773	Bacillus sonorensis SRCM101395	ganA	2055
Deinococcus sp. NW-56	rpoB	3459	Paenibacillus polymyxa CF05	ganA	1053
Sphingopyxis granuli TFA	rpmJ	126	Frankia sp. QA3	galE	1041
Saccharopolyspora erythraea DSM 40517	rpmI	195	Caldicellulosiruptor bescii RKCB131	fusA	2076
Brevibacillus laterosporus DSM 25	rpmE	201	Phaeobacter inhibens P54	fur	414
Janthinobacterium svalbardensis PAMC 27463	rpmE	270	Dyadobacter fermentans NS114, DSM 18053	fumC	1404
Lactobacillus delbrueckii bulgaricus ATCC BAA-365	rpmC	198	Aulosira laxa NIES-50	ftsH	1938
Sphingopyxis lindanitolerans WS5A3p	rpmB	294	Nitrosospira multiformis Nl4	ftsH	1896
Bifidobacterium kashiwanohense PV20-2	rplX	336	Frankia sp. QA3	FRAAL6651	570
Terriglobus roseus AB35.6	rplW	294	Frankia alni ACN14a	FRAAL2500	804
Sphaerobacter thermophilus 4ac11, DSM 20745	rplV	345	Frankia alni ACN14a	FRAAL2444	270
Candidatus Protochlamydia naegleriophila KNic	rplV	336	Frankia alni ACN14a	FRAAL1235	1128
Micromonospora aurantiaca ATCC 27029	rplR	390	Frankia alni ACN14a	FRAAL0999	912
Hydrogenobaculum sp. HO	rplQ	357	Modestobacter marinus BC501	folA	579
Luteibacter sp. 329MFSha	rplQ	387	Thermoclostridium stercorarium stercorarium DSM 8532	folA	501
Novosphingobium sp. P6W	rplP	435	Thermoclostridium stercorarium stercorarium DSM 8532	fliE	300
Corynebacterium striatum 216	rplI	453	Cupriavidus nantongensis X1	flgK	1920
Propionibacterium freudenreichii freudenreichii DSM 20271	rplE	663	Phaeobacter inhibens P92	flgC	393
Nitrosomonas sp. IS79A3	rplD	621	Priestia megaterium QM B1551	flbD	216
Solitalea canadensis USAM 9D, DSM 3403	rplD	630	Paenibacillus sp. lzh-N1	fhuD1	960
Nitrospira defluvii	rplC	621	Arthrospira platensis C1	ffh	1416
Rhodopseudomonas palustris TIE-1	RPA4706	582	Corynebacterium striatum KC-Na-01	fda	1035
Rhodopseudomonas palustris CGA009	RPA3762	873	Rhodococcus jostii DSM 44719	fadE26	1182
Rhodopseudomonas palustris TIE-1	RPA3585	513	Simkania negevensis Z, ATCC VR-1471	fabG-B	744
Rhodopseudomonas palustris CGA009	RPA2908	645	Phaeobacter inhibens P80	fabB	1230
Rhodopseudomonas palustris TIE-1	RPA2269	1803	Bacillus sp. IHB B 7164	eutC	714
Rhodopseudomonas palustris CGA009	RPA0026	1398	Phaeobacter gallaeciensis P11	edd	1824
Syntrophus gentianae DSM 8423	rny	1569	Rubrobacter radiotolerans RSPS-4	dut	456
Paenibacillus polymyxa J	rluD3	999	Laribacter hongkongensis LHGZ1	dut	450
Paenibacillus polymyxa E681	rimM	516	Pseudodesulfovibrio profundus 500–1	dsrK	1668
Xanthomonas albilineans XaFL07-1	rimK	876	Aliarcobacter butzleri NCTC 12481	dprA	777
Comamonas sp. 26	recR	591	Desulfotalea psychrophila LSv54	DP2200	201
Phaeobacter gallaeciensis P128	recR	600	Streptomyces sp. 1222.2	dnaQ2	726
Klebsiella pneumoniae pneumoniae RJF293	recF	1074	Propionibacterium freudenreichii freudenreichii DSM 20271	dnaN	1161
Phaeobacter inhibens P83	recA	1068	Frankia sp. EUN1f	dnaJ	1146
Actinoplanes sp. SE50	rbsA	1512	Frankia sp. EUN1f	dnaJ	1179
Xanthomonas albilineans HVO082	rbfA	411	Streptomyces sp. Root1310	dnaE1	3540
Nitrosococcus watsoni C-113	queA	1035	Bacillus licheniformis 9945A	dnaB	1425
Limnospira indica PCC 8005	pyrR	534	Caldithrix abyssi LF13, DSM 13497	dnaA	1401
Bacillus licheniformis DSM 13 Novozymes	pyrP	1305	Sulfurovum sp. NBC37-1	dnaA	1329
Paenibacillus polymyxa SC2	pyrC	1323	Janthinobacterium sp. 61	dksA	453
Thioalkalivibrio sp. ALJ5	pyrC	1296	Geobacter sulfurreducens PCA	divIC	336
Streptomyces sp. 1222.2	pyrAA	1143	Priestia megaterium ATCC 14581	desR	603
Streptomyces sp. 1222.2	puuC	1458	Deinococcus proteolyticus MRP, DSM 20540	der	1326
Paenibacillus polymyxa Mc5Re-14	purR11	1041	Micromonospora sp. CNZ295	deoD	708
Corynebacterium doosanense CAU 212, DSM 45436	purN	567	Actinoplanes teichomyceticus DSM 43866	deoA	1278
Lactobacillus delbrueckii lactis KCCM 34717	purL	2223	Bacillus paralicheniformis Bac84	degQ	141
Deinococcus proteolyticus MRP, DSM 20540	purK	1119	Phaeobacter piscinae P71	def1	519
Propionibacterium freudenreichii shermanii JS	purE	561	Halorhodospira halophila SL1	ddl	915
Cytophaga hutchinsonii ATCC 33406	purB	1371	Qipengyuania flava VG1	dcd	555
Paenibacillus polymyxa SC2	pucR	1665	Frankia sp. QA3	dapB	753
Phaeobacter inhibens P88	ptsP	2241	Paenibacillus polymyxa M1	dapB	804
Janthinobacterium sp. 13	pssA	852	Propionibacterium freudenreichii shermanii JS	dapB	741
Candidatus Accumulibacter regalis UW-1	psd	858	Saccharopolyspora erythraea NRRL 2338	dapA1	924
Vitis vinifera PN40024	psaJ	132	Bacillus sp. FJAT-21351	dacF	1167
Actinoplanes friuliensis DSM 7358	prsA2	981	Laribacter hongkongensis LHGZ1	cysB1	939
Desulfohalobium retbaense HR100, DSM 5692	prmA	891	Bacillus licheniformis 5NAP23	cydC	1725
Thermoanaerobacter wiegelii Rt8.B1	priA	2199	Corynebacterium glyciniphilum AJ 3170	ctaC	1113
Thioalkalivibrio sp. AKL12	prfA	1086	Xanthomonas albilineans GPE PC17	cspA2	246
Mycobacterium bovis BCG Tokyo 172	ppsC	6567	Thermoanaerobacter sp. X514	crcB	405
Modestobacter multiseptatus DSM 44402	ppiB	537	Geobacter sulfurreducens KN400	corA-2	954
Cutibacterium acnes PA_30_2_L1	PPA1529	468	Anabaenopsis circularis NIES-21	corA	1143
Cutibacterium acnes PA_30_2_L1	PPA0469	1068	Priestia megaterium DSM 319	comGF	438
Cutibacterium acnes AE1	PPA0083	2217	Priestia megaterium WSH-002	comGB	1047
Paenibacillus polymyxa Mc5Re-14	potD1	1074	Phaeobacter inhibens P78	codA	1281
Priestia megaterium ATCC 14581	ponA	2835	Salinispora pacifica DSM 45543	cobD	1002
Xanthomonas albilineans GPE PC17	pntA-2	318	Frankia alni ACN14a	coaX	753
Candidatus Saccharimonas aalborgensis	pnp	2124	Corynebacterium variabile DSM 44702	cmk	657
Actinoplanes sp. SE50	pks3A	2271	Deferribacter desulfuricans SSM1	clpX	1233
Actinoplanes sp. SE50/110	phy1	1215	Bifidobacterium actinocoloniiforme DSM 22766	clpP	633
Paenibacillus polymyxa CICC 10580	phnX	837	Arthrospira platensis YZ	chlL	867
Nostoc sp. PCC 7120	phnD	1002	Desulfovibrio cf. magneticus IFRC170	cheW	477
Desulfosudis oleivorans Hxd3	pheT	2412	Corynebacterium glutamicum ATCC 21831	Cgl3047	156
Cylindrospermum stagnale PCC 7417	pheT	2436	Corynebacterium glutamicum ATCC 21831	Cgl2611	1485
Janthinobacterium sp. 67	pgsA	585	Corynebacterium flavum ZL-1	Cgl2418	252
Xanthomonas albilineans GPE PC86	pglA	1173	Corynebacterium flavum ZL-1	Cgl2255	294
Priestia megaterium DSM 319	pfyP	645	Corynebacterium flavum ZL-1	Cgl1238	1143
Verrucosispora sp. CNZ293	pfp	1029	Corynebacterium glutamicum ATCC 21831	Cgl1220	1146
Propionibacterium freudenreichii freudenreichii DSM 20271	pf456	1536	Corynebacterium glutamicum ATCC 13869	Cgl1195	564
Pseudodesulfovibrio indicus J2	pcm	642	Corynebacterium crudilactis JZ16	Cgl0754	681
Candidatus Protochlamydia amoebophila UWE25	pc0987	336	Corynebacterium glutamicum ATCC 13869	Cgl0388	1833
Candidatus Protochlamydia amoebophila UWE25	pc0116	1773	Corynebacterium flavum ZL-1	Cgl0337	642
Bacillus sp. 1 s-1	parC	2424	Actinoplanes sp. SE50	celA	1506
Dakarella massiliensis ND3	parC	2556	Cupriavidus taiwanensis LMG 19424	cca	1248
Micromonospora sp. L5	parA	924	Thermoanaerobacter kivui DSM 2030	carB	3219
Xanthomonas albilineans PNG130	panD	381	Bradyrhizobium sachari BR 10556	bll7821	2097
Thioalkalivibrio paradoxus ARh 1	panC	855	Bacillus paralicheniformis Bac84	BLi02578	438
Fibrella aestuarina BUZ 2	pacB	432	Bifidobacterium longum longum CCUG30698	BL0873	1596
Rhodococcus opacus ATCC 51882	paaN	2040	Bifidobacterium longum longum CCUG30698	BL0422	1977
Collimonas arenae Ter10	paaF	777	Bacillus sp. 1 s-1	BL03510	618
Streptomyces scabiei 87.22	oppD3	996	Bacillus licheniformis 5NAP23	BL03504	1314
Bacillus sonorensis SRCM101395	oppD	1068	Bacillus licheniformis 5NAP23	BL03493	2433
Mycoplasmoides fermentans M64	oppC-1	999	Bifidobacterium longum longum CCUG30698	BL0349	627
Paenibacillus polymyxa Sb3-1	occM1	660	Bacillus paralicheniformis ATCC 12759	BL03105	543
Rubrobacter radiotolerans RSPS-4	nusG	540	Bacillus paralicheniformis 14DA11	BL02837	1857
Xanthomonas sacchari LMG 476	nusG	558	Bacillus sp. H15-1	BL02416	660
Delftia sp. GW456-R20	nuoN	1494	Bacillus paralicheniformis 14DA11	BL01721	1995
Phaeobacter gallaeciensis P129	nuoM	1548	Bacillus licheniformis 5NAP23	BL01149	327
Phaeobacter inhibens P92	nuoL	2130	Bacillus sonorensis SRCM101395	BL00226	393
Thioalkalivibrio paradoxus ARh 1	nuoI	489	Paenibacillus polymyxa CF05	bioB	1008
Kribbella flavida IFO 14399, DSM 17836	nuoD	1206	Xanthomonas translucens pv. cerealis CFBP 2541	bfr	471
Acidovorax sp. 93	nuoD	1254	Lelliottia nimipressuralis SGAir0187	betA	1665
Isoptericola variabilis 225	nuoA	363	Frateuria aurantia Kondo 67, DSM 6220	bamB	1209
Rhodococcus koreensis DSM 44498	nuoA	360	Thauera chlorobenzoica 3CB1	azo1431	852
Streptomyces sp. 57	nucS	672	Saccharopolyspora erythraea NRRL 2338	atzB	1392
Sphingopyxis sp. C-1	nodQ	1902	Delftia sp. 60	atpH	540
Nitrospira defluvii	NIDE4034	333	Thioalkalivibrio sp. ALgr1	atpH	537
Nitrospira defluvii	NIDE1341	573	E. coli 2886–75	atpG	795
Cupriavidus metallidurans CH34	nemA	1110	Nitrosococcus watsoni C-113	atpG	870
Geobacillus thermocatenulatus KCTC 3921	ndoA	351	Leuconostoc gelidum gasicomitatum LMG 18811	atpC	450
Phaeobacter inhibens P80	ndk	423	Phaeobacter gallaeciensis P129	atpC	414
Flavobacterium johnsoniae UW101, ATCC 17061	nbaC	540	Nostoc sp. PCC 7107	asr1559	252
Streptomyces sp. 57	nagB	786	Nostoc sp. PCC 7107	asr0064	237
Fimbriimonas ginsengisoli Gsoil 348	nadK	849	Xanthomonas albilineans MTQ032	aspS	1752
Nitrosospira briensis Nsp8	nadA	1101	Geobacillus sp. C56-T3	aroA	1083
Paenibacillus polymyxa M1	arnT	2355	Micromonospora sp. CNZ295	alaS	2679
Bacillus paralicheniformis MDJK30	argJ	1221	Acidovorax sp. 93	ahcY	1434
Sphaerobacter thermophilus 4ac11, DSM 20745	argH	1371	Modestobacter marinus BC501	adk	624
Janthinobacterium svalbardensis PAMC 27463	argA	1320	Lactobacillus delbrueckii bulgaricus ND02	addA	3684
Ligilactobacillus salivarius salivarius UCC118	apt	519	Collimonas arenae Ter282	aceK	1785
Priestia megaterium QM B1551	amt	1227	Lysinibacillus sp. YS11	accA	957
Nostoc sp. PCC 7120	alr4917	1689	Cupriavidus necator NH9	aat	759
Nostoc sp. PCC 7120	alr3663	1050	Phaeobacter inhibens P88	aat	633
Nostoc sp. PCC 7120	alr2594	435	Nostoc sp. PCC 7120	all4101	384
Trichormus variabilis ATCC 29413	alr0203	480	Nostoc sp. Moss5	all3116	738
Nostoc sp. PCC 7120	all5344	468	Nostoc sp. PCC 7120	all1863	864
Trichormus variabilis NIES-23	all4824	798	Trichormus variabilis ATCC 29413	all0781	1590
			Trichormus variabilis ATCC 29413	all4426	1254

### Pathway analysis

**Fig. 10:**
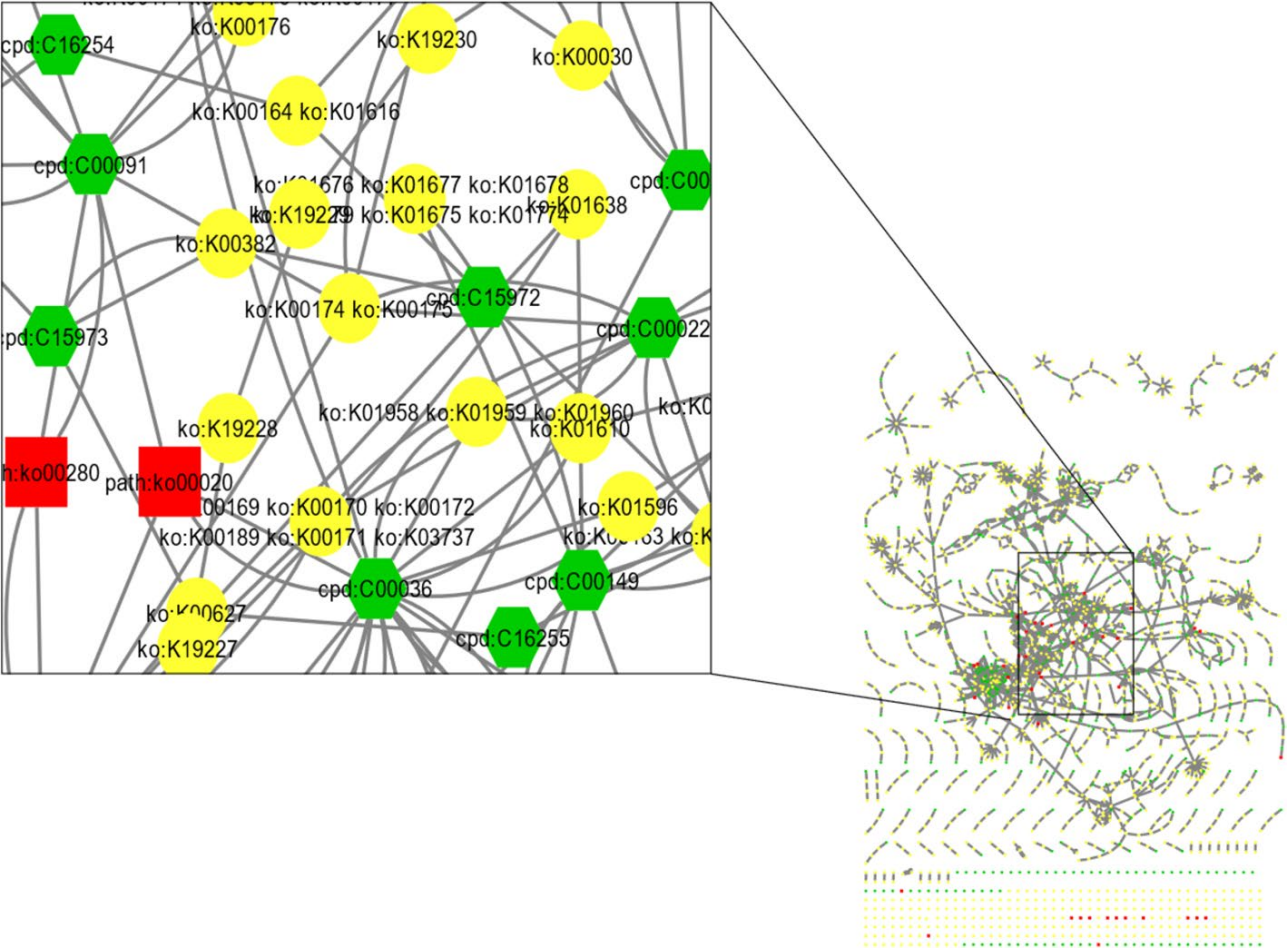
The Pathway Enrichment analysis

Pathway analysis is a powerful tool to understand the biological significance of gene lists generated from high-throughput experiments. In this study, pathway analysis was used to identify 16 significant pathways enriched in the predicted genes. The network of pathway enrichment of the metagenome data has been shown in Fig. 10. These pathways are involved in a variety of essential cellular processes, including biosynthesis, energy production, and signaling. The CMP-KDO biosynthesis II (from D-arabinose 5-phosphate) pathway is one of the most significant pathways identified in this study [39]. It is involved in the biosynthesis of lipopolysaccharide (LPS), an essential component of the outer membrane of gram-negative bacteria (Fig. 1S(i)). Two sequences, aconitate hydratase (K01681) and citrate synthase (K01647), are associated with the TCA cycle pathway. They have been found in Glycine max (soybeans) and Saccharomyces cerevisiae (yeast). The TCA cycle is essential for optimal functioning of primary carbon metabolism in plants (Fig. 1S(ii-iv)). Aconitate hydratase catalyzes the isomerization of citrate to isocitrate in the TCA cycle. The function of aconitate hydratase has been well studied in model plants, such as Arabidopsis thaliana. The TCA cycle is a metabolic process that occurs in plants, animals, fungi, and other bacteria. It is a series of chemical reactions that converts acetyl-CoA into carbon dioxide and energy. The TCA cycle is an important source of energy for cells and also plays a role in the synthesis of other molecules such as amino acids and fatty acids [40]. The next pathway involved the biosynthesis of fatty acids (Fig. 1S(v)). This is essential for the formation of membranes, which are necessary for the viability of all cells, except Archaea. Fatty acids are also a compact energy source for seed germination. Enenoyl-[acyl-carrier protein] reductase I (K00208) is an enzyme involved in fatty acid biosynthesis, prodigiosin biosynthesis, and biotin metabolism pathways. Another significant pathway that has been identified is biotin metabolism (Fig. 1S(vi)). The Biotin metabolism is a universal and essential process that is required for intermediary metabolism in all three domains of life: archaea, bacteria, and eukaryotes [41]. It is an indispensable vitamin for human health and plays a vital role in well-being [42]. This essential nutrient can be obtained through the consumption of a wide range of foods, including legumes, soybeans, tomatoes, romaine lettuce, eggs, cow's milk, and oats. One of the primary functions of biotin is to act as a cofactor for enzymes, facilitating carboxylation reactions that are crucial for processes, such as gluconeogenesis, amino acid catabolism, and fatty acid metabolism. In addition, it produces biochemicals that have a wide variety of applications in nutrition and industry. Another gene sequence, also been discovered that encodes a protein called 2-dehydro-3-deoxyphosphooctonate aldolase (K01627), also known as Kdo-8-phosphate synthase (KDO 8-P). This protein is a major constituent of the outer leaflet of the outer membrane of most Gram-negative bacteria. It is essential for the survival of bacteria and pathogens, and is involved in the biosynthesis of nucleotide sugars (Fig. 1S(vii)) and lipopolysaccharide biosynthesis pathways (Fig. 1S(viii)) [43]. Another important pathway has been discovered that is essential for plant growth and development that is called the porphyrin and chlorophyll metabolism pathway (Fig. 1S(ix)). Porphyrins are a group of organic compounds essential for life. They are found in chlorophyll, which is a green pigment that plants use for photosynthesis. Porphyrins are also found in heme, a protein that carries oxygen in the blood. The porphyrin and chlorophyll metabolic pathways are complex processes that involve the synthesis of porphyrins and chlorophyll. This pathway is essential for plant growth and development because it provides plants with the materials required to produce chlorophyll and heme. The porphyrin and chlorophyll metabolic pathways are synthesized by a multistep pathway that involves eight enzymes [44], which is a complex process involving numerous chemical reactions catalyzed by enzymes. The regulation of chlorophyll and heme balance is important for the growth and development of plants [45]. Porphyrin biosynthesis is one of the most conserved pathways known, with the same sequence of reactions occurring in all species. By associating different metals, porphyrins give rise to the “pigments of life”: chlorophyll, heme, and cobalamin [46]. The glyoxylate and dicarboxylate metabolic pathways play a pivotal role in the sustenance of organisms and their biochemical functions (Fig. 1S(x)). In the glyoxylate pathway, citrate synthase (K01647) is responsible for citrate formation from acetyl-CoA and oxaloacetate. Citrate is then converted into succinate, which is used to synthesize glucose [47]. This process entails the conversion and application of these intermediates, which are generated via the catabolism of fatty acids, metabolism of amino acids, and fermentation of carbohydrates. These compounds can facilitate the synthesis of diverse molecules such as glucose, amino acids, and fatty acids. The glyoxylate and dicarboxylate pathway holds significant importance in the realm of plant physiology, owing to the fact that unlike animals, plants are unable to stockpile carbohydrates in the form of glycogen. Rather than undergoing direct utilization, fatty acids are transformed into glucose molecules, which play a crucial role in supporting growth and reproduction. The bacterial pathway is of paramount importance, as it facilitates the conversion of carbon dioxide into organic compounds, thereby enabling the acquisition of energy from carbon dioxide. In general, glyoxylate and dicarboxylate pathways are crucial and intricate metabolic pathways that are indispensable for the viability of diverse organisms. The aforementioned metabolic pathway is important for the generation of energy, carbon metabolism, and production of biomolecules. Comprehending the overall metabolic network and its implications in cellular function requires a thorough understanding of the intricacies of glyoxylate and dicarboxylate metabolism. The process of prodigiosin biosynthesis was initially characterized in the γ-proteobacterium, Serratia marcescens. Subsequently, it was studied and characterized in another bacterium, Pseudoalteromonas rubra. In these organisms, prodigiosin biosynthesis involves a series of biochemical reactions and enzymatic steps that lead to the production of this captivating red pigment (Fig. 1S(xi)). By exploring the biosynthetic pathways of prodigiosin in different bacterial species, researchers have gained valuable insights into the diversity and complexity of this intriguing pigment and its potential applications in various fields. ABC transporters pathway are a large, ancient protein superfamily found in all living organisms (Fig. 1S(xii)). They function as molecular machines by coupling ATP binding, hydrolysis, and phosphate release for the translocation of diverse substrates across membranes. ABC transporters are also known as efflux pumps because they mediate the cross-membrane transportation of various endo- and xenobiotic molecules energized by ATP hydrolysis [48] and the arginine transport system substrate-binding protein(K09996), which specifically binds to arginine molecules and facilitates their transport across the cell membrane. This protein is part of a large complex involved in cellular arginine uptake. Substrate-binding proteins play a crucial role in the arginine transport system by recognizing and capturing arginine molecules from the extracellular environment and initiating their transport into cells. It ensures the specificity and efficiency of arginine uptake, contributing to various biological processes that require arginine as a nutrient or signaling molecule. The biosynthetic pathways of cofactors employ a greater quantity of innovative organic chemistry compared to other pathways in primary metabolism (Fig. 1S(j)). As a result, there is a wealth of research being conducted on the mechanisms of cofactor biosynthetic enzymes [49]. There are two sequence of arginine transport system substrate-binding protein (ASBP) (K09996, K09997) have been associated with biosynthetic pathways of cofactors. The function of these protein is unknow. It is a protein that is involved in the transport of arginine across the cytoplasmic membrane of bacteria. ASBP is a member of the ABC transporter family, which is a large family of proteins that are involved in the transport of a variety of molecules across membranes [50]. The small subunit ribosomal protein S12 (K02950) is present in both mitochondrial and bacterial ribosomes (Fig. 1S(xiv)). Ribosomal protein S12 is an essential component of the small subunit within the ribosome that is responsible for protein synthesis. In E. coli, S12 plays a significant role in facilitating translation initiation. This protein consists of approximately 120–150 amino acid residues and is fundamentally basic in nature [51]. Two-component systems represent intricate signaling pathways that have gained significant attention during the initial stages of the 1980s. Their emergence into the spotlight can be primarily attributed to their identification within the paradigmatic microorganism, E. coli (Fig. 1S(xv)). These systems provide living organisms with the ability to detect and convert a diverse array of incoming signals, enabling them to adjust and respond in a highly adaptable manner to alterations in both their external and internal environments [52]. Methyl-accepting chemotaxis proteins (K03406) are the most common receptors in bacteria and archaea. They are arranged as trimers of dimers that form hexagonal arrays in the cytoplasmic membrane or cytoplasm [53]. Methyl-accepting chemotaxis proteins (MCPs) are also involved in bacterial chemotaxis, which is essential for the host colonization and virulence of many pathogenic bacteria causing human, animal, and plant diseases (Fig. 1S(xvi)). These receptors undergo reversible methylation during the adaptation of the bacterial cells to environmental attractants and repellents. They are also involved in bacterial chemotaxis. MCPs are concentrated at the cell poles in an evolutionarily diverse panel of bacteria and archae [54]. They are classified into different classes according to their ligand-binding region and membrane topology. Chemotaxis is the process by which cells sense chemical gradients in their environment and move towards more favorable conditions. MCPs are a family of bacterial receptors that mediate chemotaxis to diverse signals and respond to changes [53] (Table 4).

**Table 4:** Provides more specific breakdown of information on pathways and sequences

Pathway	Species	#Seqs	KO
CMP-KDO biosynthesis II (from D-arabinose 5-phosphate)	Arabidopsis lyrate	k141_2112_1	AT1G53000.1
TCA cycle (plant)			K01681
Fatty acid biosynthesis	None	k141_2161_1	K00208
Citrate cycle (TCA cycle)	None	k141_1768_1	K01647
Biotin metabolism	None	k141_2161_1	K00208
Lipopolysaccharide biosynthesis	None	k141_2112_1	K01627
Porphyrin and chlorophyll metabolism	None	k141_566_2	K00230
		k141_503_2	
Glyoxylate and dicarboxylate metabolism	None	k141_1768_1	K01647
Prodigiosin biosynthesis	None	k141_2161_1	K00208
ABC transporters	None	k141_1767_1	K09996
Biosynthesis of cofactors	None	k141_566_2	K09996
		k141_503_2	K09997
		k141_2161_1	
Ribosome	None	k141_588_1	K02950
		k141_2090_1	
		k141_1643_1	
		k141_1118_1	
		k141_1964_1	
		k141_2047_1	
		k141_1682_1	
Two-component system	None	k141_1278_1 s	K03406
Biosynthesis of nucleotide sugars	None		K01627
Bacterial chemotaxis	None		K03406
Citric acid cycle (TCA cycle)	Saccharomyces cerevisiae	k141_1768_1	K01647

## Conclusion

The enormous amount of data generated from omics studies is possible only with fast and accurate handy omics tools, as they are available on today’s scientific platform. Metagenomics is an area that is booming with the advent of NGS. Metagenomics is the study of the genes and genomes of microbes that cannot be cultured in a laboratory. Metagenomic data of a sample can be generated by shotgun sequencing of total community DNA. The gene sequences obtained from metagenomic shotgun sequencing can be Nucleotide-Nucleotide BLAST (blastn) mapped to the available microbial genomes in public domain databases, such as NCBI GenBank, RefSeq, and Integrated Microbial Genomes (IMG). This will provide an inventory of all microbial genera and species present in the sample. The unmapped reads were annotated using different in silico tools, such as DIAMOND, KEGG, CAZy, and eggNOG, for the identification of genes and their functions. Taxonomic and functional annotation of genes helps understand the metabolic pathways that are unique to a particular microbiome. Based on these inferences, it is possible to propose models for the role of microbes in health and disease. Soybean is one of the most important crops in the world and a major source of protein and oil. The soybean endosphere is a unique microenvironment colonized by a large number of bacteria, fungi, and viruses. The composition of the endospheric microbiome differs from that of the rhizospheric microbiome. The microbiome of the endosphere plays an important role in plant health. In this study, we aimed to elucidate the usefulness of the microbiome in revealing the signatures of microbes in healthy and diseased soybean agricultural lands. To identify the microbes associated with health and disease, microbial diversity in the soybean endosphere was analyzed by metagenomic analysis using the MG-RAST tool. The analysis of the soybean endosphere microbiome revealed signatures of microbes associated with health and disease. The most dominant group of bacteria in the endosphere is Streptomyces, followed by Chryseobacterium, Paenibacillus, Bacillus, and Mitsuaria. These bacteria play a role in a variety of biological pathways, including CMP-KDO biosynthesis II (from D-arabinose 5-phosphate), TCA cycle (plant), citrate cycle (TCA cycle), fatty acid biosynthesis, and glyoxylate and dicarboxylate metabolism. These data revealed that it is a rich source of potential biomarkers for soybean plants. The results of this study will help us understand the role of the endosphere microbiome in plant health and identify the microbial signatures of health and disease.

### Supplementary Information

**Additional file 1: Figure S1. **Pathway analysis.

**Additional file 2: Table S1.**

## Data Availability

In the Manuscript.
