# Supplementary material for: Metagenomic analysis of soybean endosphere microbiome to reveal signatures of microbes for health and disease
Source: J Genet Eng Biotechnol. 2023 Aug 16;21:84. doi: 10.1186/s43141-023-00535-4 (PMC10429481; doi:10.1186/s43141-023-00535-4)
Supplement: Supplementary file 1 — Additional file 1: Figure S1. Pathway analysis. [file 43141_2023_535_MOESM1_ESM.docx]

**Supplementary Figures**

Figure S1. Pathway analysis


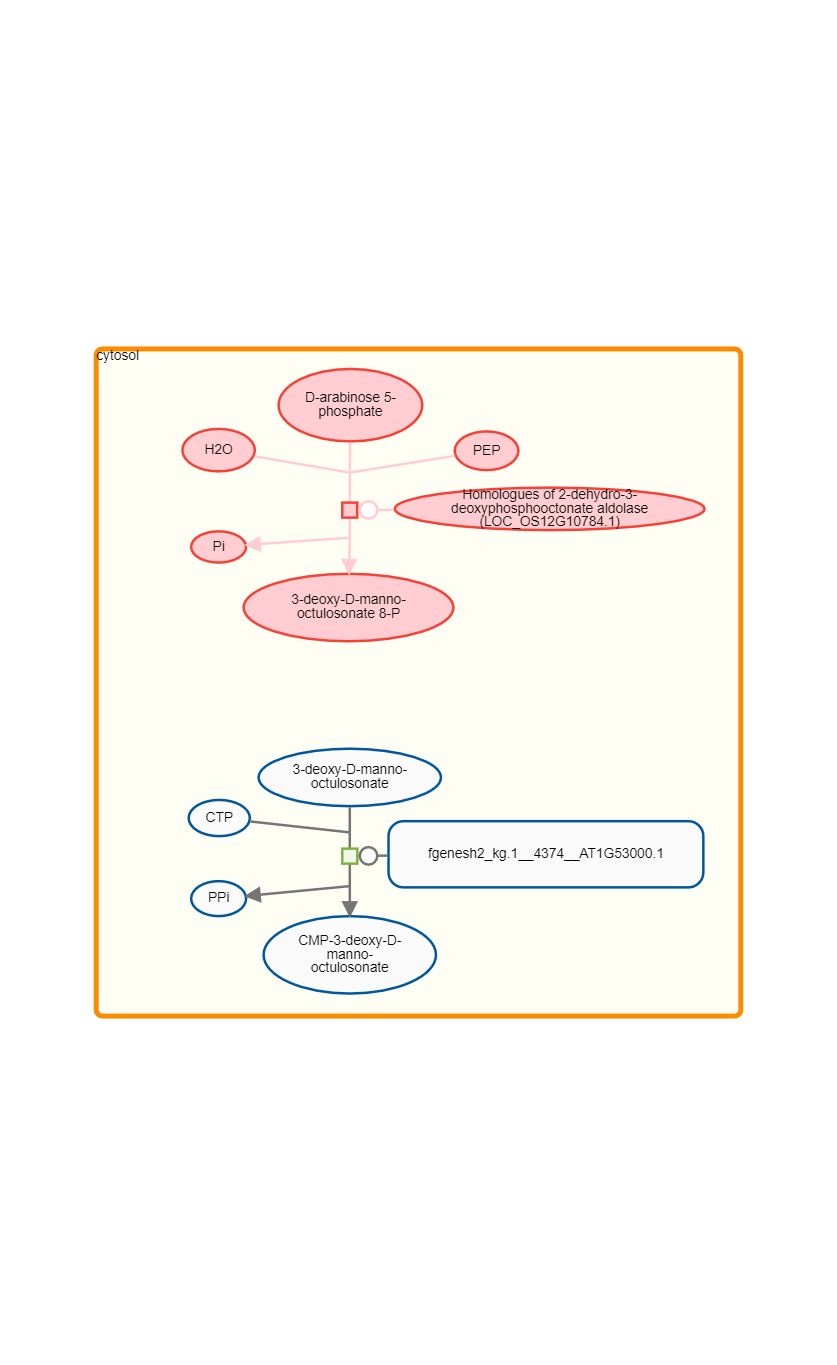


(i) CMP-KDO biosynthesis II (from D-arabinose 5-phosphate)


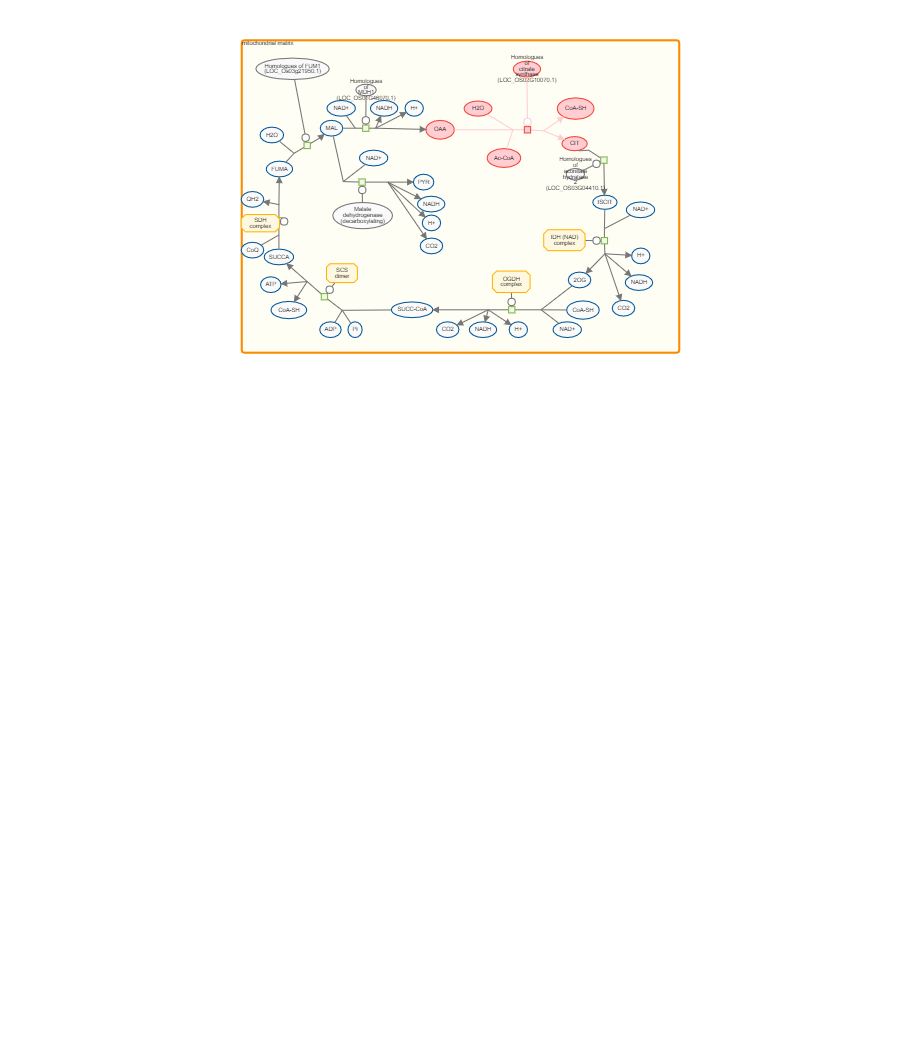


(ii) TCA cycle (plant)


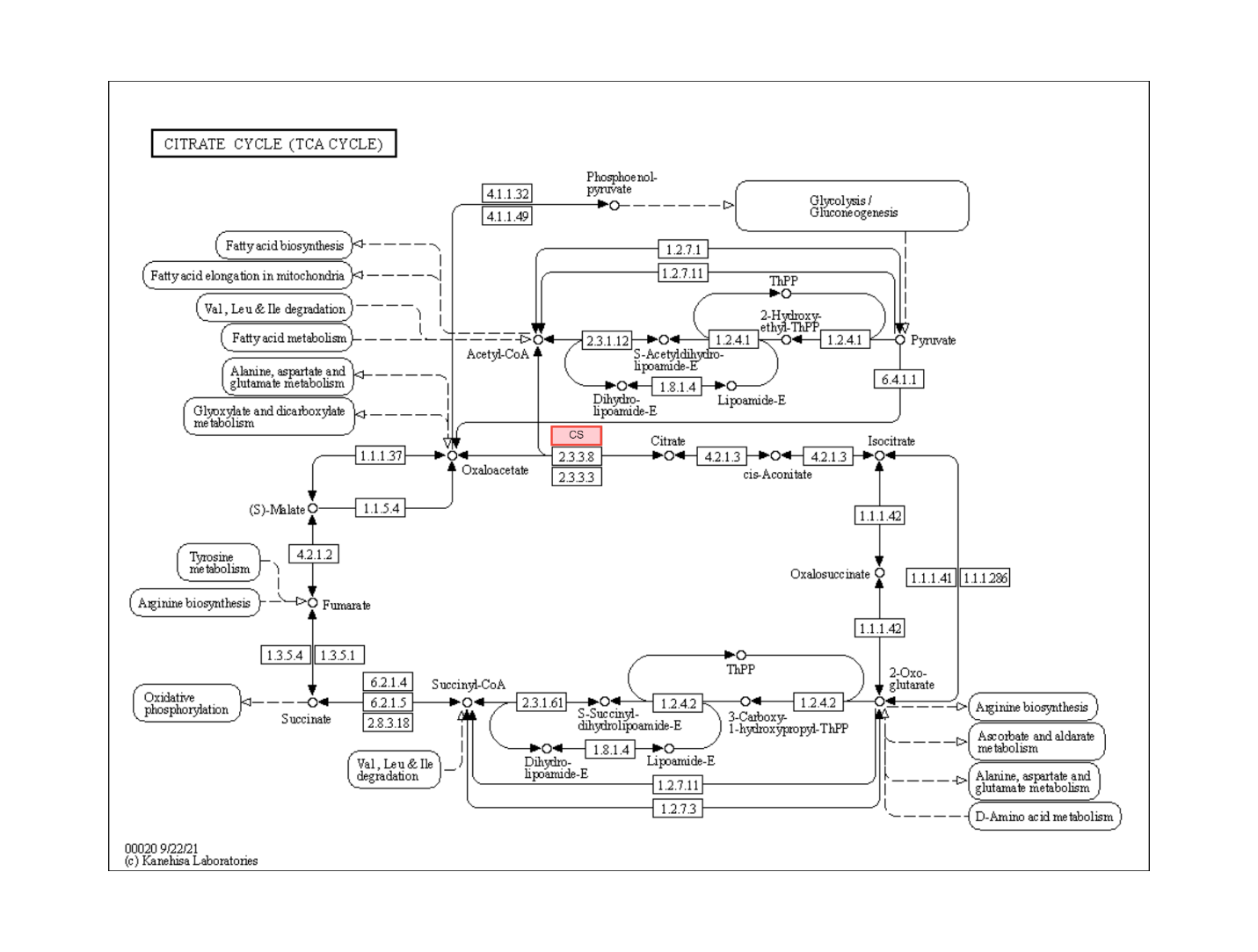


(iii) Citrate cycle (TCA cycle)


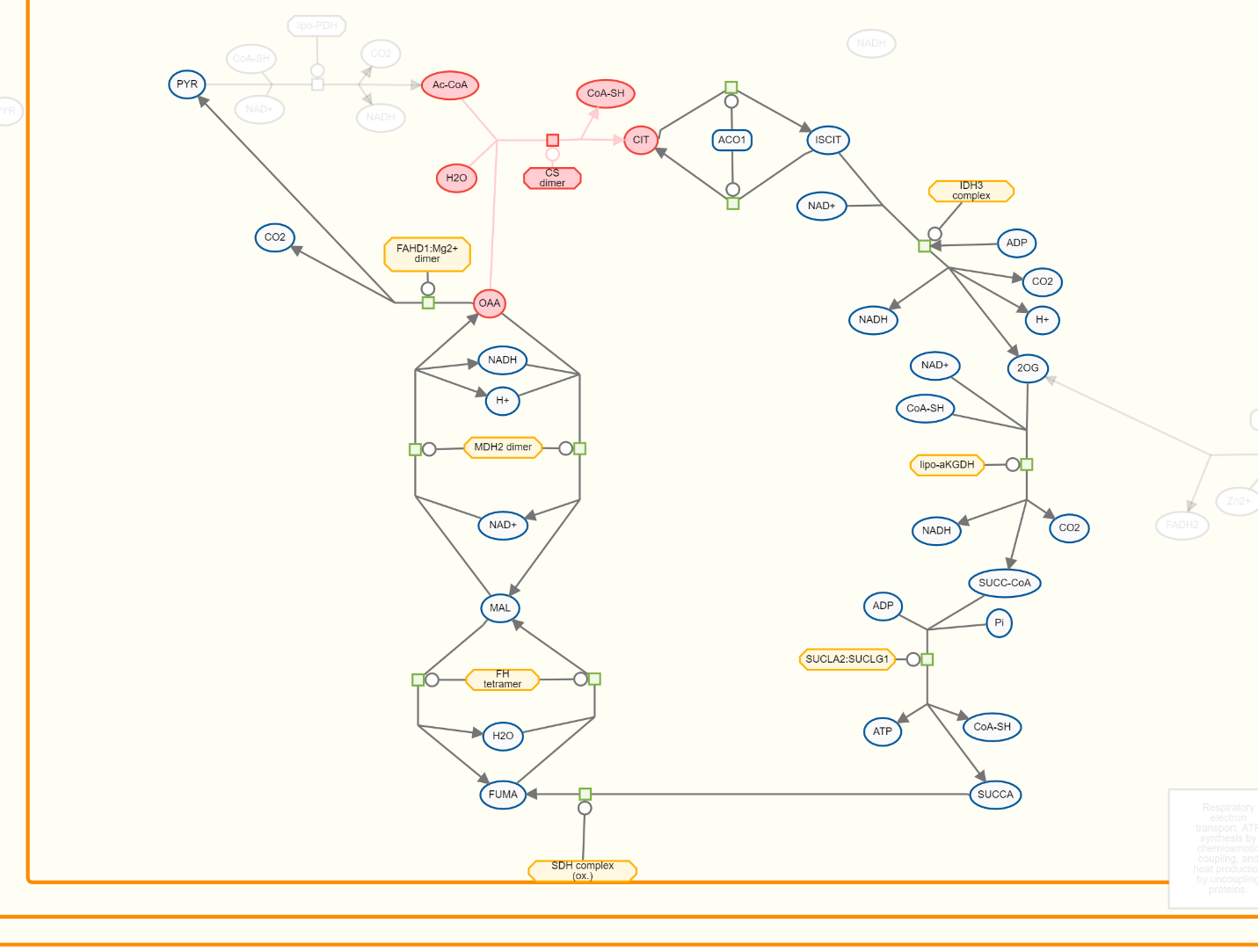


(iv) Citric acid cycle (TCA cycle)


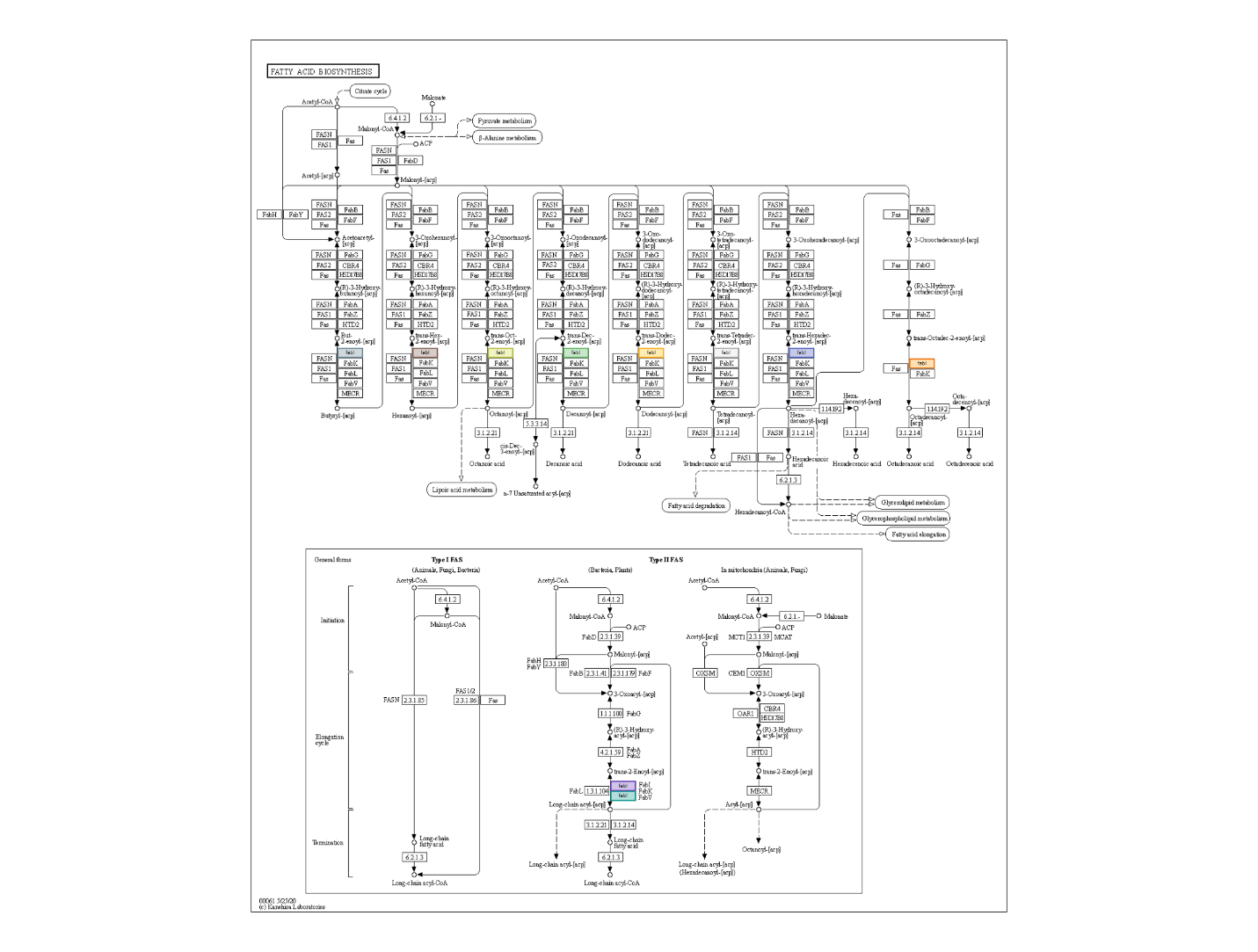


(v) Fatty acid biosynthesis


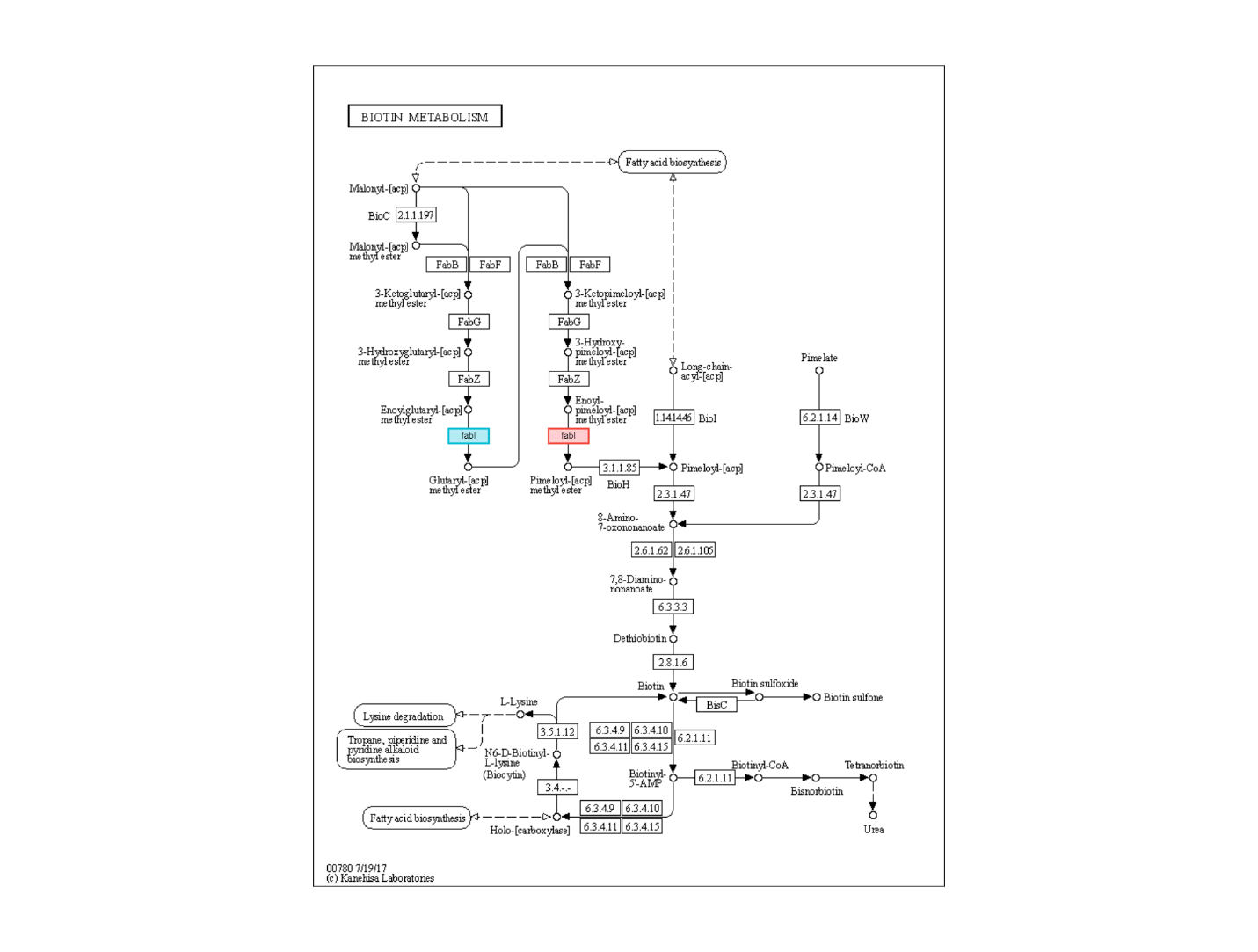


(vi) Biotin metabolism
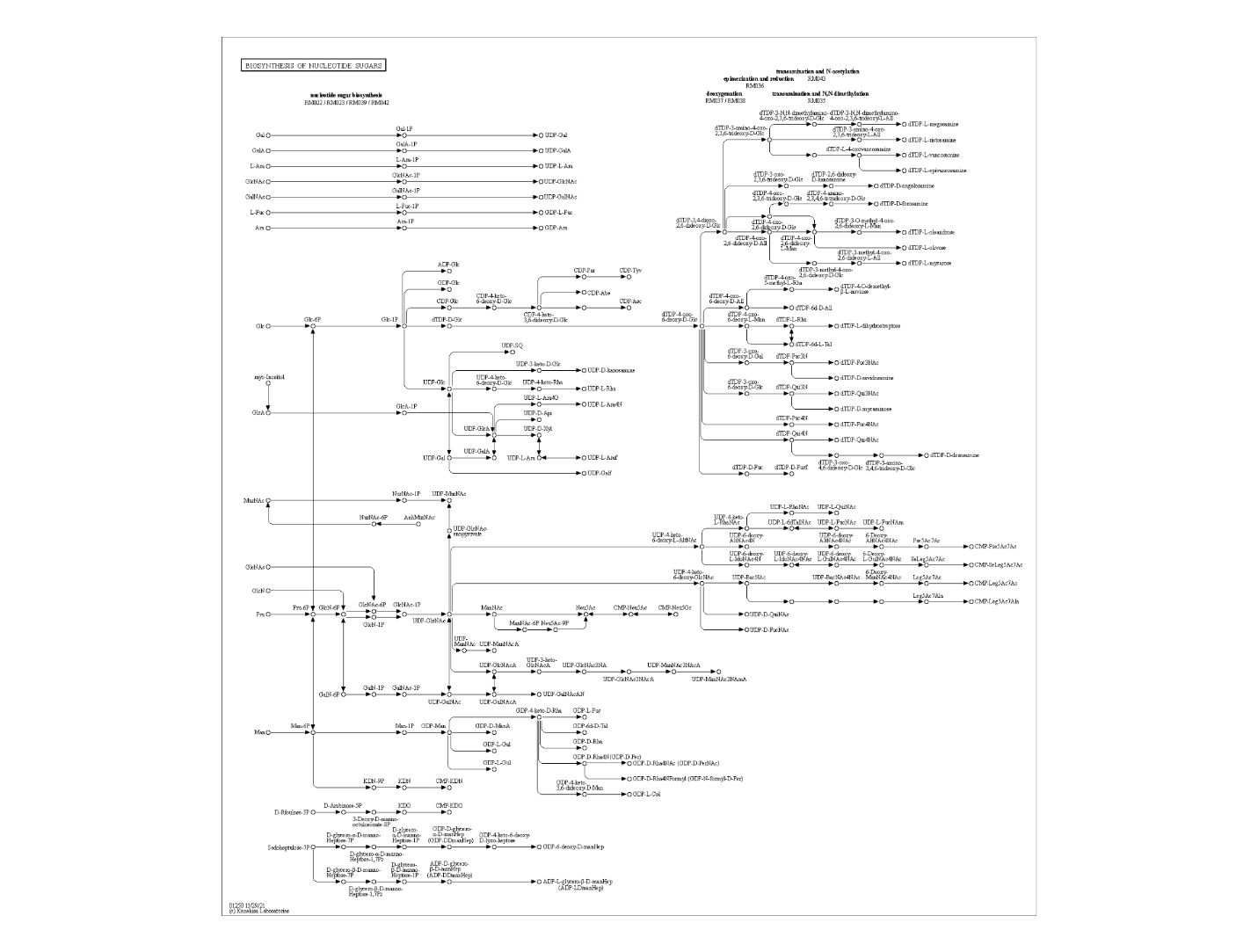


(vii) Biosynthesis of nucleotide sugars


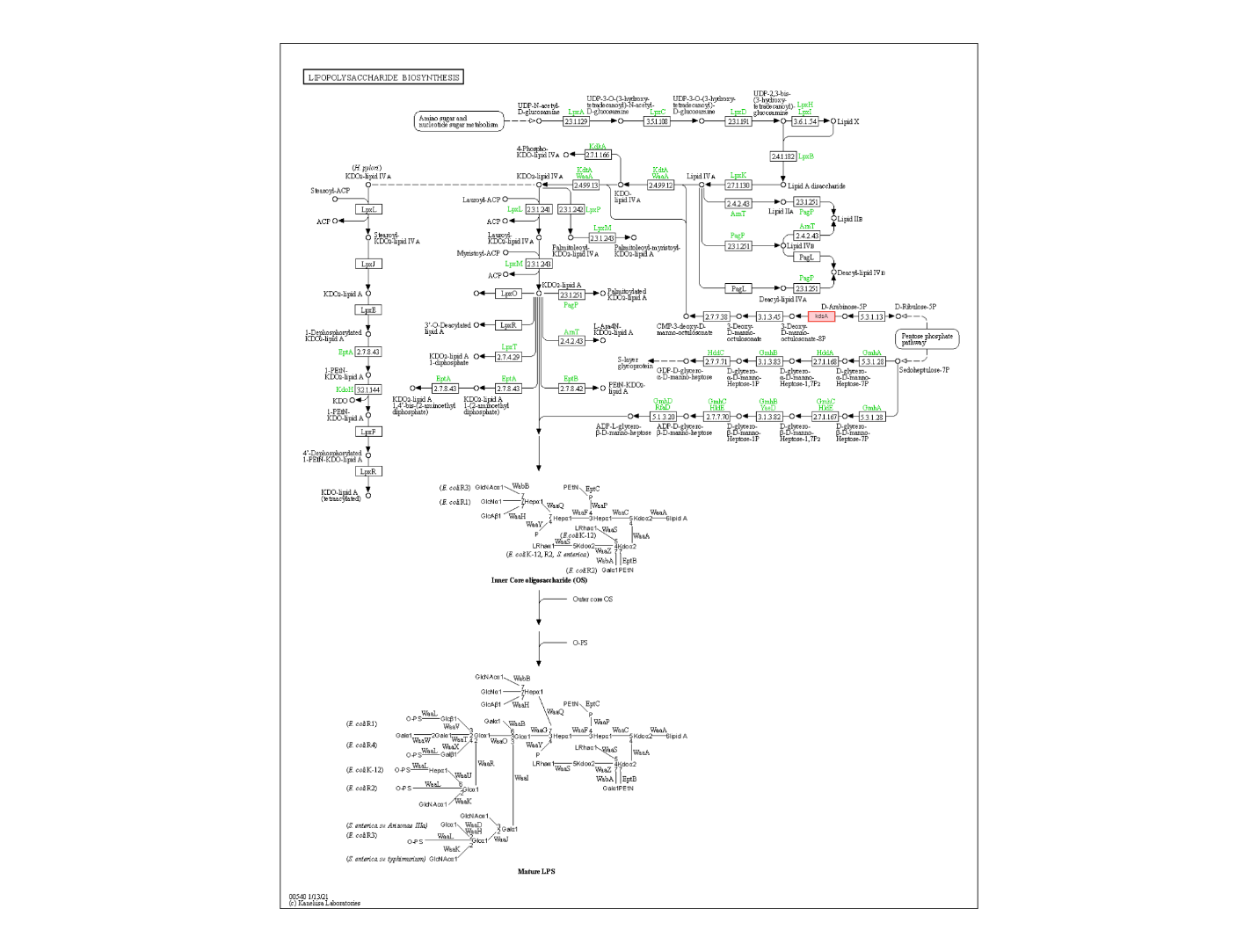


(viii) Lipopolysaccharide biosynthesis


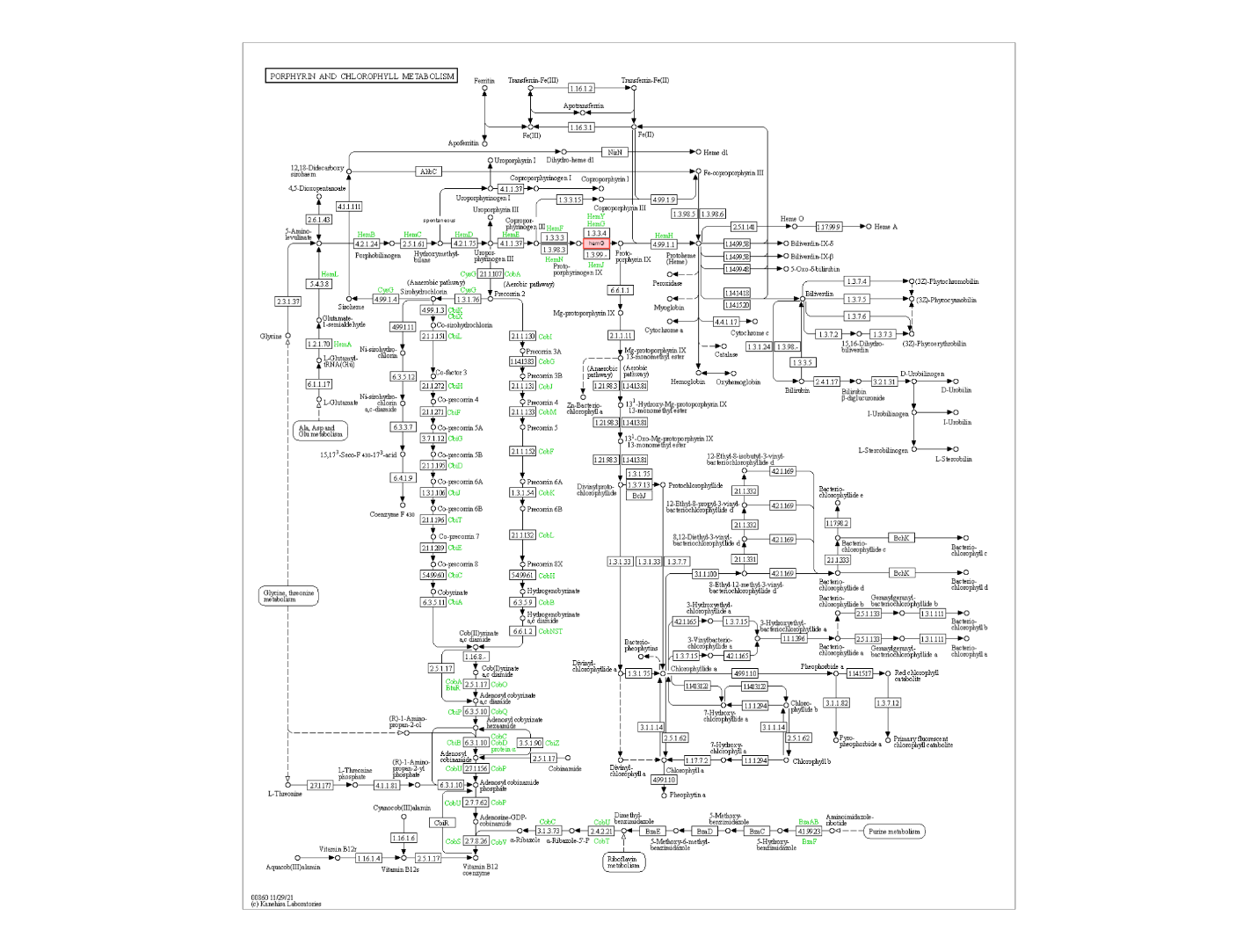


(ix) Porphyrin and chlorophyll metabolism


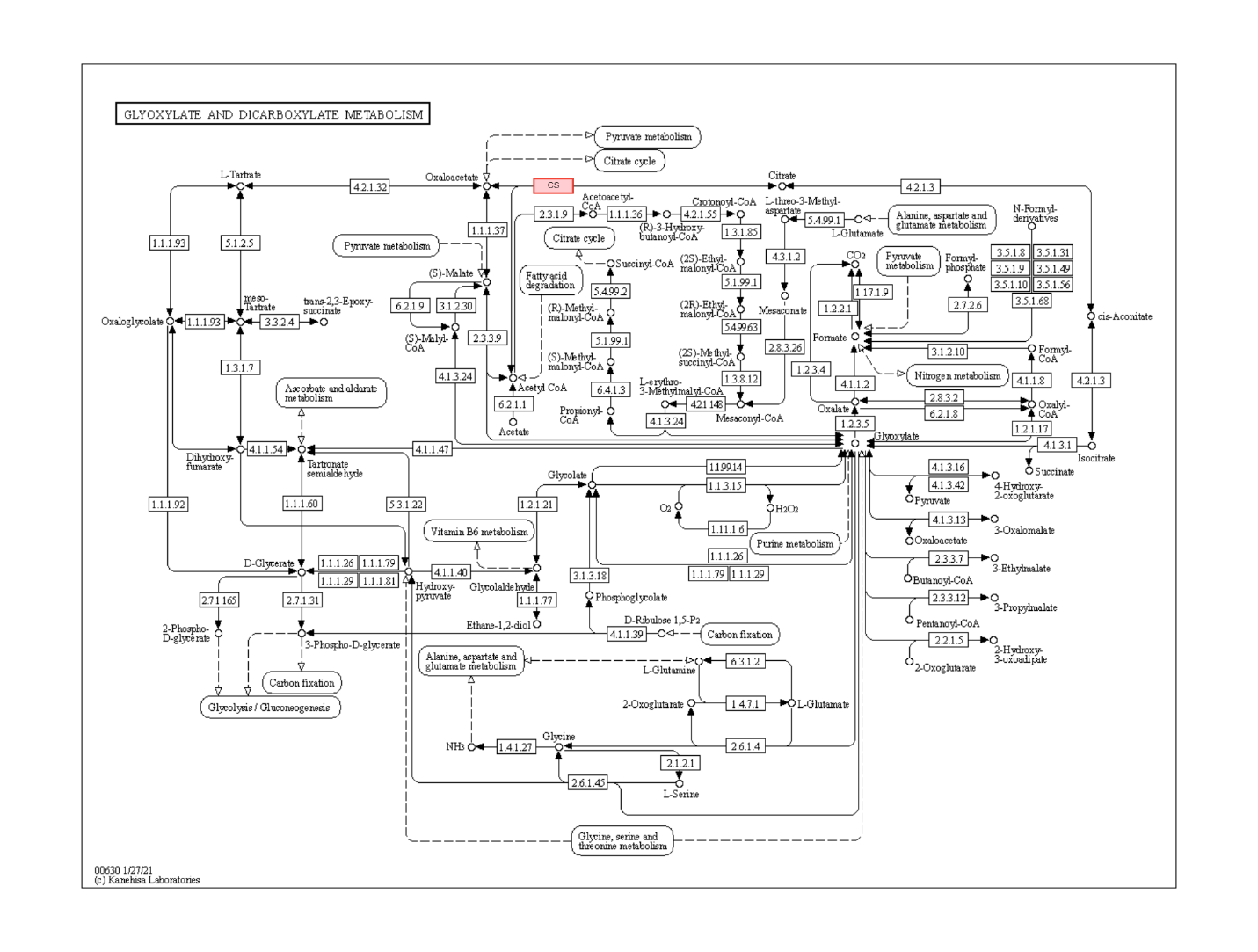


(x) Glyoxylate and dicarboxylate metabolism


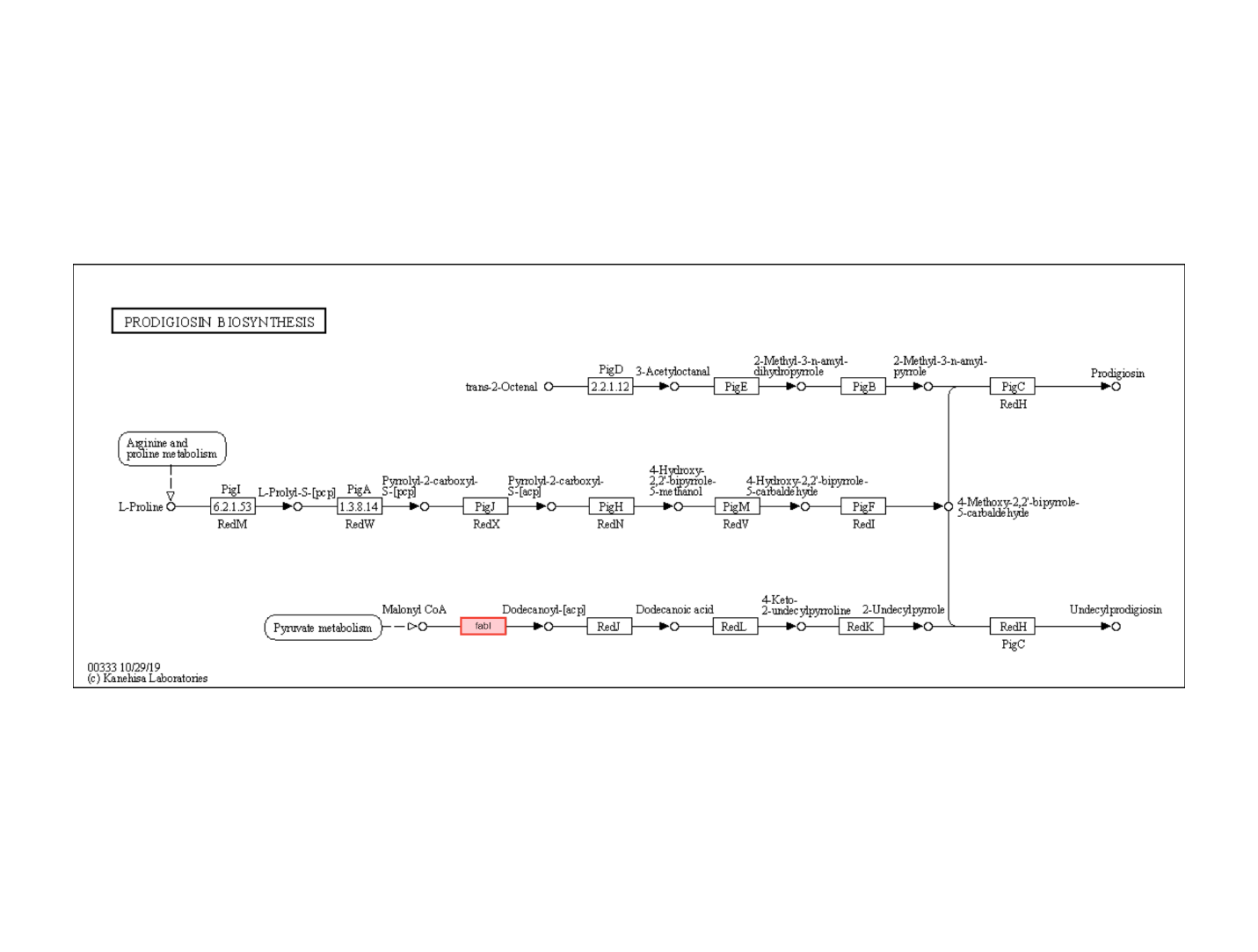


1. Prodigiosin biosynthesis


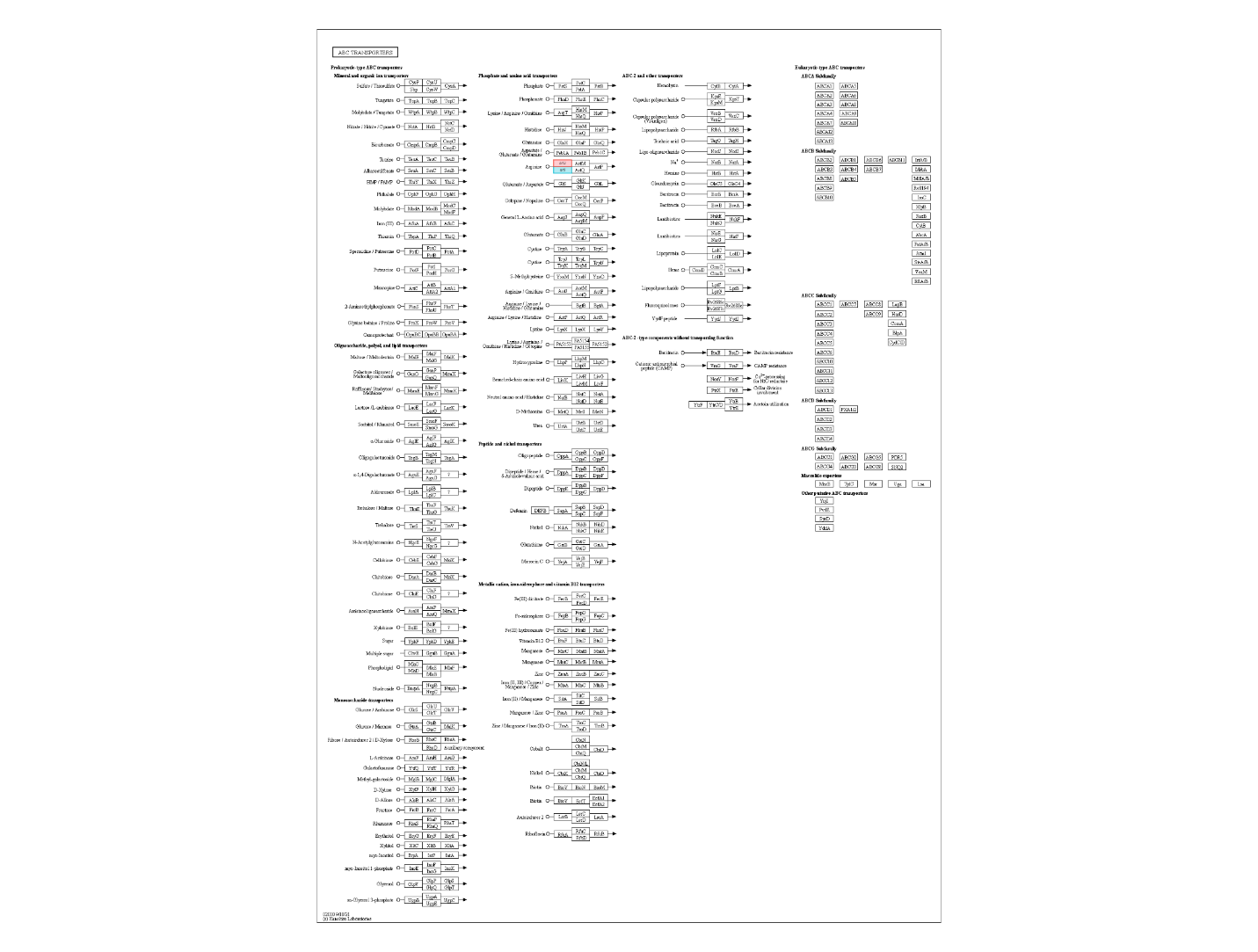


(xii) ABC transporters


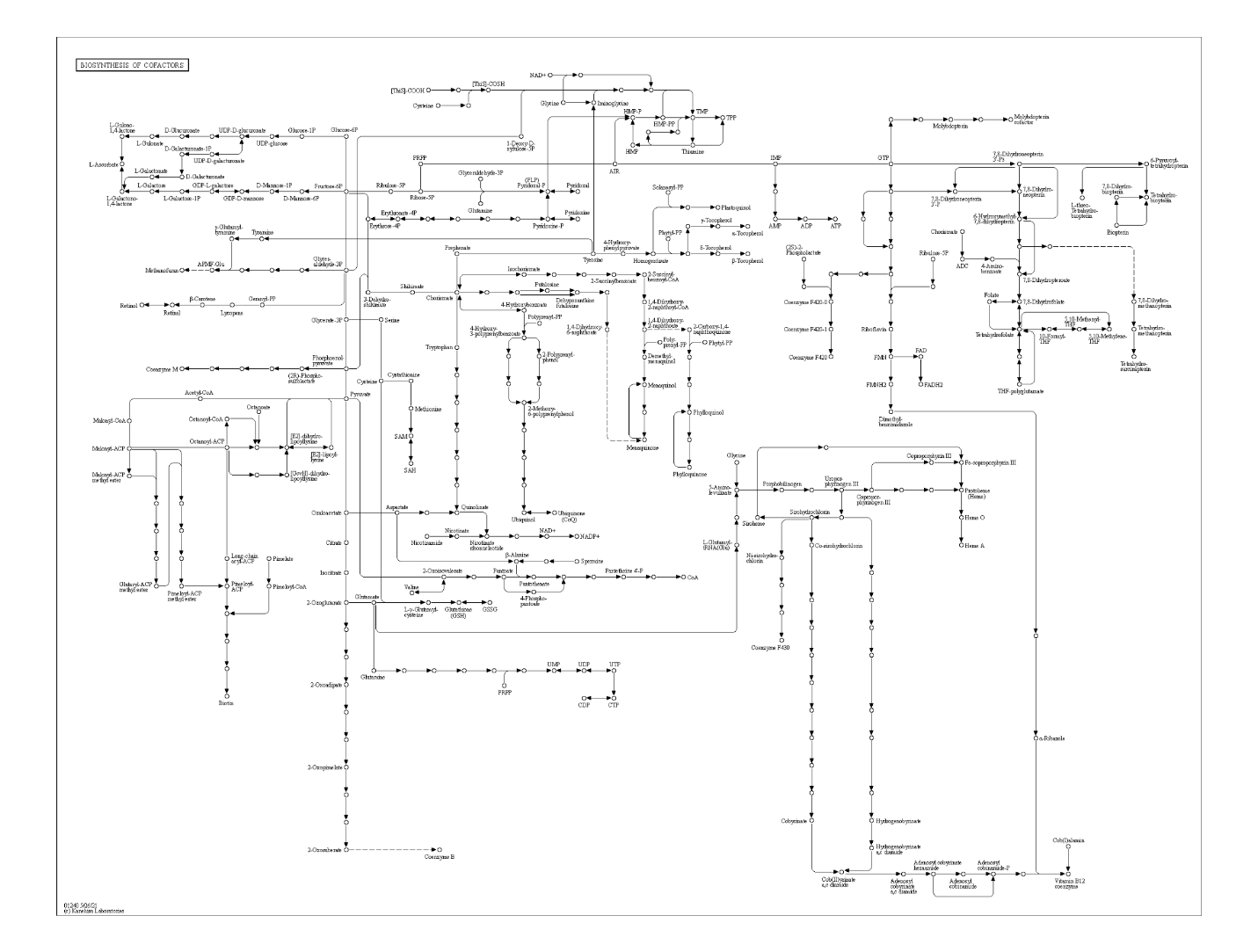


(xiii) Biosynthesis of cofactors


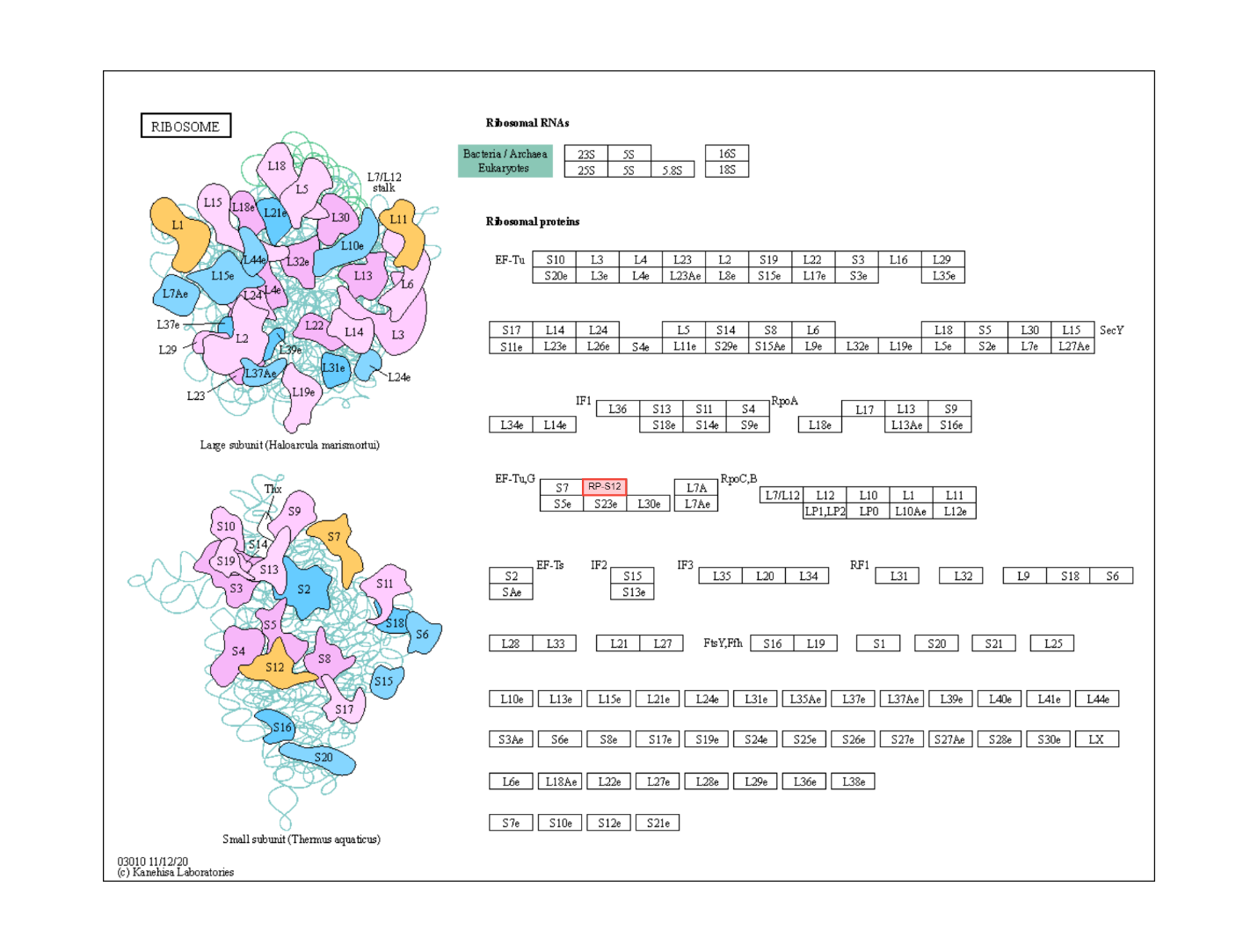


(xiv) Ribosome


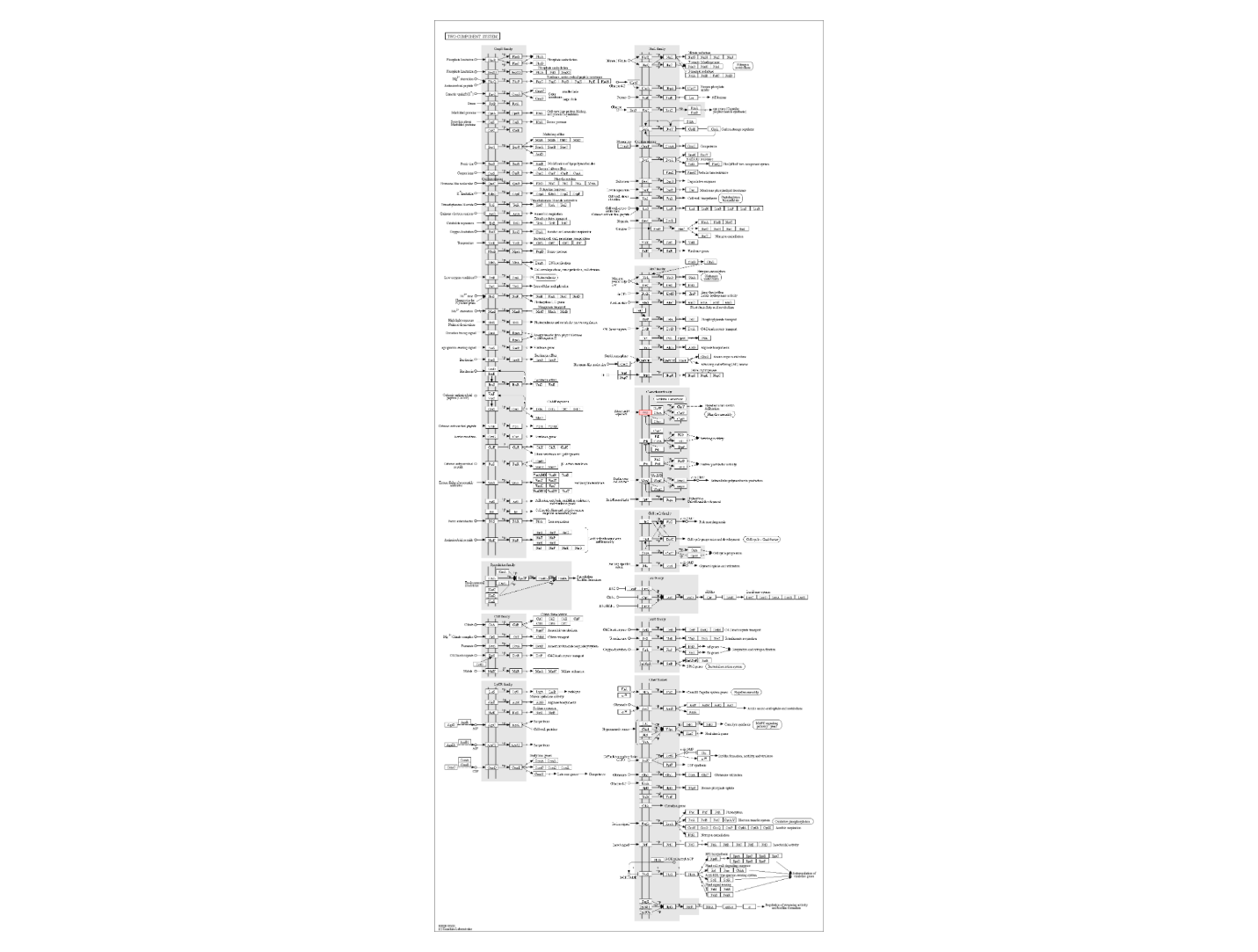


(xv) Two-component system


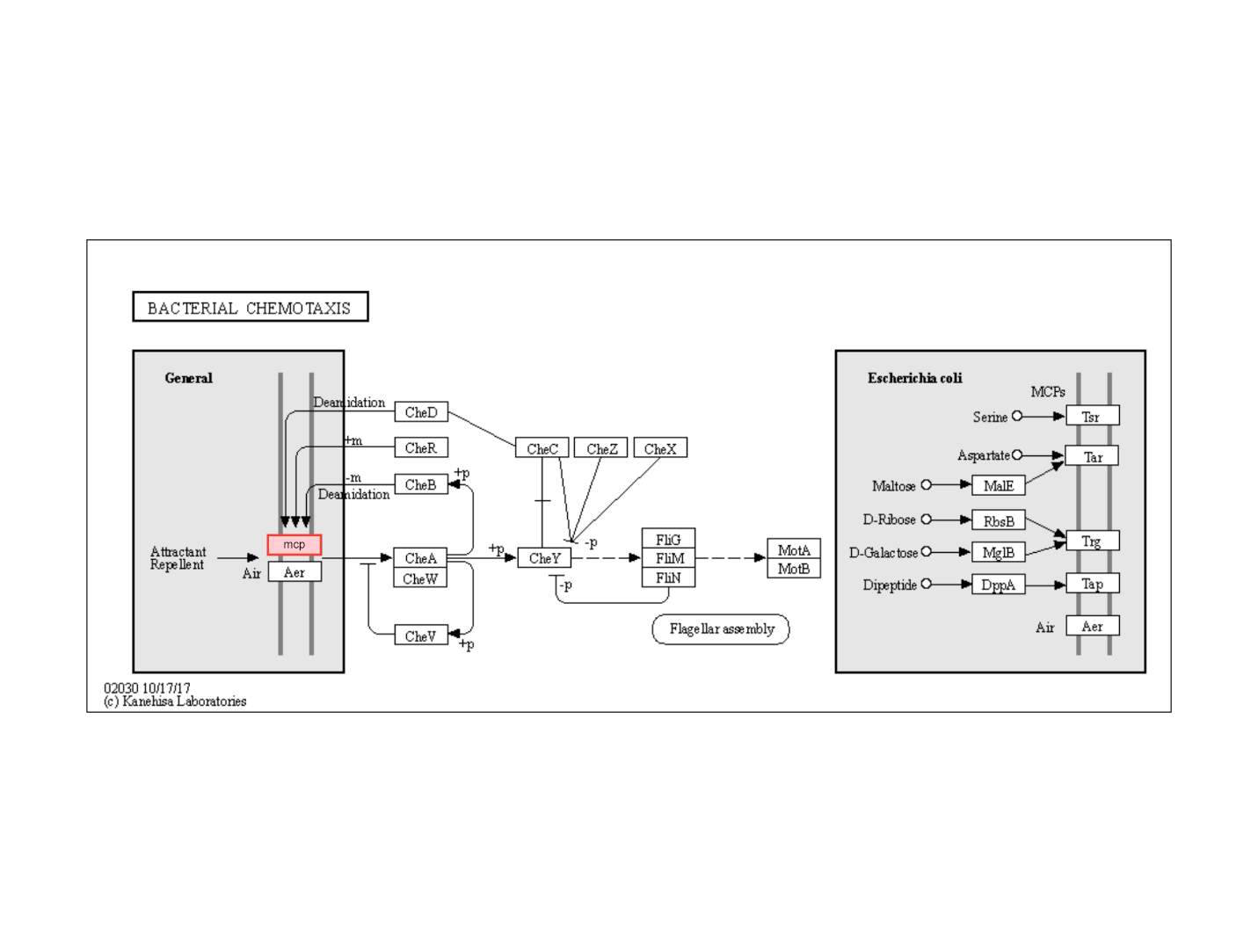


(xvi) Bacterial chemotaxis

((

(

(
